# Identifying Food Packaging Migrants: Current Analytical Capabilities, Challenges, and Future Prospects

**DOI:** 10.1111/1541-4337.70474

**Published:** 2026-04-09

**Authors:** Xue‐Chao Song, Qi‐Zhi Su, Elena Canellas, Qin‐Bao Lin, Yu Zhou, Cristina Nerin

**Affiliations:** ^1^ School of Food and Nutrition Anhui Agricultural University Hefei Anhui China; ^2^ Guangzhou Customs Technology Center Guangzhou Guangdong China; ^3^ Department of Analytical Chemistry, Aragon Institute of Engineering Research I3A, EINA University of Zaragoza Zaragoza Spain; ^4^ College of Packaging Engineering Jinan University Zhuhai Guangdong China

**Keywords:** food packaging materials, high‐resolution mass spectrometry, identification, migration, nontargeted analysis

## Abstract

The migration of intentionally and non‐intentionally added substances (IAS/NIAS) from food packaging into foodstuffs presents a significant challenge to consumer health and food safety. Accurate and comprehensive identification of these chemical migrants is therefore paramount. This review systematically summarizes recent advances in the analytical workflows used to identify these migrants. We critically evaluate the latest developments in both gas chromatography coupled to mass spectrometry (GC–MS) and liquid chromatography coupled to high‐resolution mass spectrometry (LC–HRMS). Special attention is given to cutting‐edge techniques, such as comprehensive two‐dimensional gas chromatography (GC × GC) for enhanced separation of complex mixtures, high‐resolution filtering (HRF) for leveraging the dual advantages of gas chromatography coupled to high‐resolution mass spectrometry (GC–HRMS) accurate mass measurements and conventional low‐resolution spectral matching, and ion mobility spectrometry (IMS) for its unique ability to resolve isomers. Concurrently, we provide an in‐depth critique of the evolving data analysis strategies, from conventional targeted analysis to the more comprehensive suspect and nontargeted screening approaches. The principles, advantages, and limitations of each workflow are discussed in the context of their application to food packaging materials. Then, the review dissects major bottlenecks, notably the scarcity of reference standards and comprehensive mass spectral libraries, which hinder confident identification. Looking forward, we highlight promising future directions, emphasizing that the synergistic integration of open‐access mass spectral databases, adoption of novel analytical techniques, and machine learning‐based molecular property prediction will facilitate the identification of IAS and NIAS in food packaging. In addition, integrating chemical analysis with bioassays will enable the prioritization of high‐hazard chemicals, ultimately improving the safety evaluation of food packaging.

## 1 Introduction

Food packaging materials serve the crucial function of protecting food from microbial contamination and mechanical damage throughout the supply chain, encompassing transportation, distribution, storage, retail, handling, and consumer use. A diverse range of materials is utilized for this purpose, including plastic, paper and cardboard, glass, metal, and ceramic (Muncke 2016). Compared to inert materials like glass, metal, and ceramic, plastics, paper, and cardboard are noninert; these materials are more susceptible to chemical migration into the contacting food and the surrounding environment due to their inherent permeability and the presence of low‐molecular‐weight compounds (Gupta et al. 2024).

Thousands of chemicals, known as intentionally added substances (IAS), are utilized in the manufacture of food packaging and other food contact materials (FCMs) (Groh et al. 2019; Groh et al. 2021). Generally, IAS are incorporated to enhance the performance or extend the service life of the final product. The most common IAS in food packaging materials include plasticizers, antioxidants, flame retardants, light and heat stabilizers, slip agents, and lubricants (Geyer et al. 2017). In addition to IAS, non‐intentionally added substances (NIAS) are also formed within these materials during all stages of production, use, and end of life, as a result of side reactions, degradation, or contamination (Nerin et al. 2013; Nerin et al. 2022).

Both IAS and NIAS can migrate from food packaging materials into contained food products, posing potential risk for human health (Alberto Lopes and Tsochatzis 2023; Alberto Lopes et al. 2021; Arvanitoyannis and Bosnea 2004; Aznar et al. 2019; Bignardi et al. 2017; da Silva Oliveira et al. 2019). Recent evidence indicates that many food contact chemicals (FCCs) have already been detected in humans (Geueke et al. 2024), and their effects at current exposure levels are relevant for human health (Landrigan et al. 2025; Muncke et al. 2020; Parkinson et al. 2024; Symeonides et al. 2024). For instance, studies by Wagner and colleagues demonstrated that chemicals commonly found in plastic food packaging, such as phthalates, organophosphate esters (OPEs), and fatty acids, can disrupt endocrine function and metabolism (Stevens et al. 2024; Volker et al. 2022; Zimmermann et al. 2021; Zimmermann et al. 2019). Furthermore, Zhang et al. (2017) identified fluorene‐9‐bisphenol, a bisphenol A (BPA) alternative used in plastic water bottles, as an antiestrogenic agent capable of disrupting pregnancy in mice. This toxicological concern is further exemplified by the European Food Safety Authority's (EFSA) recent decision to lower the tolerable daily intake (TDI) for BPA to a level significantly below current analytical capacity (EFSA et al. 2023). Such a stringent regulatory shift highlights the critical necessity for advancements in sensitive identification and quantification strategies. NIAS also present health concerns; exposure to 2,4‐di‐tert‐butylphenol, a degradation product of the antioxidant Irgafos 168, was shown to increase lipid accumulation by activating Retinoid X Receptors (Ren et al. 2023). Given the potential health risks posed by both IAS and NIAS, elucidating their structures and characterizing their occurrence in diverse food packaging materials remains crucial.

Gas chromatography–mass spectrometry (GC–MS) and liquid chromatography–mass spectrometry (LC–MS) are the primary analytical platforms for detecting migrants from food packaging materials, generally suited for volatile/nonpolar and nonvolatile/polar compounds, respectively, although their application scopes overlap (e.g., phthalate plasticizers can be analyzed by both) (Jia et al. 2014; Otero et al. 2015). While previous reviews have discussed the identification of IAS and NIAS and the capabilities of various techniques (Kato and Conte‐Junior 2021; Nerin et al. 2013; Nerin et al. 2022; Wrona and Nerin 2020), recent advancements, particularly the rise of high‐resolution mass spectrometry (HRMS), sophisticated data processing software, and expanding tandem mass spectra (MS/MS) databases, have catalyzed a significant shift from purely targeted analysis toward comprehensive suspect screening and nontargeted analysis (NTA). Building upon this evolving landscape, this review extends beyond conventional methodologies to critically evaluate emerging analytical techniques for detecting IAS and NIAS in food packaging, systematically comparing their merits and limitations. We examine the roles, advantages, and limitations of traditional targeted analysis alongside the increasingly prevalent suspect screening and NTA workflows. Finally, current bottlenecks and future prospects in the field are discussed. We hope this review can advance the reliable structural elucidation of migrated compounds from food packaging materials, thereby supporting evidence‐based safety assessment and regulatory decision‐making.

## 2 IAS and NIAS in Food Packaging

Approximately 12,000 IAS may be utilized in the manufacture of food packaging materials (Groh et al. 2021). Based on their functional and structural characteristics, these IAS can be broadly categorized into four classes: functional additives, colorants, fillers, and reinforcements (Hahladakis et al. 2018). Functional additives, such as plasticizers, flame retardants, antioxidants, photoinitiators, slip agents, lubricants, and biocides, are incorporated primarily to improve the physicochemical properties of the polymer. Colorants, including pigments and soluble azo dyes, are often used in printing inks. Fillers like mica, talc, kaolin, clay, and calcium carbonate typically aim to enhance coating properties. Reinforcements, such as glass or carbon fibers, are incorporated into plastic matrices to substantially enhance their mechanical performance and structural integrity. Among these IAS classes, functional additives and colorants are the most frequently detected migrants from food packaging materials using GC–MS or LC–MS techniques (Hahladakis et al. 2018).

NIAS can form in food packaging during manufacture, use, and recycling as a result of side reactions, degradation, or contamination (Nerin et al. 2013). Side reaction products may arise either from reactions between starting substances or from reactions between migrating compounds and food components (Canellas, Vera, Nerin, et al. 2021). Oligomers, resulting from incomplete polymerization, represent a prevalent type of side reaction product found in plastic packaging. Degradation products constitute another major NIAS category. Although polymer and additive degradation occurs throughout the material's service life, external factors such as ultraviolet radiation (Yang et al. 2016) and microwave heating (Alin and Hakkarainen 2011) can significantly accelerate this process. A common example is the degradation of antioxidants. Sterically hindered phenolic antioxidants, such as butylated hydroxytoluene (BHT) and Irganox 1010, scavenge free radicals by donating hydrogen atoms, concurrently converting the phenols to quinoid structures (Beißmann et al. 2013). Secondary organophosphite antioxidants (OPAs) (e.g., Irgafos 168, Irgafos 126) reduce hydroperoxides to alcohols, undergoing transformation into phosphates themselves (Liu and Mabury 2019). Contamination is also an important type of NIAS, which becomes particularly relevant when food packaging incorporates recycled materials. Inefficient cleaning can allow various NIAS to persist, including residues from previously packaged food and environmental contamination. Furthermore, unexpected chemicals can be introduced through consumer misuse prior to recycling, where food‐grade containers are utilized to store nonfood items such as automotive fluids, pesticides, or household detergents. If the subsequent recycling process lacks a sufficiently rigorous decontamination step, these chemicals can persist in the recycled resin (Nerin et al. 2013; Nerin et al. 2022). For instance, Su, Vera, Nerin, et al. (2021) detected three pesticides (biopermethrin, chlorpyrifos, bifenthrin) in recycled high‐density polyethylene (HDPE) intended for food contact, suggesting potential cross‐contamination from agricultural plastics or consumer misuse of food‐grade HDPE for storing pesticides.

Since the primary focus of this review is to introduce emerging analytical techniques and novel data processing workflows for identifying food packaging migrants, a detailed discussion of different types of IAS and NIAS in food packaging is beyond its scope. Readers are referred to specialized literature for comprehensive coverage of these topics (Hahladakis et al. 2018; Kato and Conte‐Junior 2021; Nerin et al. 2013).

The selection of an appropriate analytical platform is fundamentally governed by the physicochemical attributes of the migrants, notably their volatility, polarity, and molecular weight, as well as the requisite detection limits. To facilitate a strategic overview prior to the detailed discussion of individual methodologies, Table 1 evaluates the performance characteristics of the primary analytical workflows utilized in food packaging research. This comparison underscores the critical trade‐offs between chemical coverage, sensitivity, and the propensity for false negatives across various GC and LC configurations. By establishing these benchmarks, the subsequent sections examine the technical capabilities and recent advancements of each platform within the context of targeted analysis and NTA.

**TABLE 1:** Comprehensive comparison of current analytical platforms for the screening of FCM migrants.

Analytical technique	Sensitivity level/LOD magnitude	Chemical coverage	False negative risk	Main strengths	Main weaknesses
Static HS–GC–MS	Moderate (µg/kg to mg/kg)	Highly volatile VOCs	High: Limited by partition coefficients and sensitivity	Simple, robust, avoids nonvolatile matrix interference	Poor sensitivity for semivolatiles
P&T–GC–MS	Excellent (ng/kg to sub‐µg/kg)	Highly volatile VOCs	Low (for VOCs): Exhaustive extraction of volatiles	Exceptional sensitivity for trace‐level volatiles	Restricted to volatiles
SPME–GC–MS	High (ng/kg to µg/kg)	Volatiles and semivolatiles	Moderate: Biased by fiber coating selectivity	Solvent‐free, high enrichment factor, fully automated	Competitive adsorption and matrix effects on fiber
GC × GC–MS	High (ng/kg to µg/kg)	Volatiles and semivolatiles	Low: Massive peak capacity; resolves co‐elutions	Superior resolution; structured elution patterns for homologous series	High data complexity; demanding method development
LC–HRMS	High (ng/kg to µg/kg)	Polar, nonvolatile, large molecules	Moderate: Ion suppression; DDA may overlook low‐intensity ions	High‐resolution accurate mass; retrospective screening capability	Matrix effects (suppression/enhancement); lacks a universal MS/MS spectra library
LC–IMS–HRMS	High (ng/kg to µg/kg)	Polar, nonvolatile, large molecules	Low: Additional IMS dimension filters interfering ions, improving identification confidence	Separation of spatial isomers; generates cleaner MS/MS spectra	Lacks of comprehensive CCS and MS/MS spectra databases; complex data interpretation

Abbreviations: CCS, collision cross section; DDA, data‐dependent acquisition; GC × GC–MS, comprehensive two‐dimensional gas chromatography coupled to mass spectrometry; LC–HRMS, liquid chromatography coupled to high‐resolution mass spectrometry; LC–IMS–HRMS, liquid chromatography coupled to ion mobility spectrometry and high‐resolution mass spectrometry; LODs, limits of detection; MS/MS, tandem mass spectra; P&T–GC–MS, purge and trap coupled to gas chromatography and mass spectrometry; SPME–GC–MS, solid‐phase microextraction coupled to gas chromatography and mass spectrometry; Static HS–GC–MS, static headspace coupled to gas chromatography and mass spectrometry; VOCs, volatile organic chemicals.

## 3 GC–MS Utilized in the Analysis of IAS and NIAS

### 3.1 One‐Dimensional (1D) GC Equipped With a Single Quadrupole System

Conventional GC–MS, that is, 1D GC equipped with a single quadrupole mass analyzer, is widely used to analyze volatile and semivolatile compounds in FCMs. In addition to its reliability and cost‐effectiveness, another advantage of a single quadrupole GC–MS system is the highly reproducible mass spectra generated by electron ionization (EI) at 70 eV, which enable reliable compound identification through spectral library matching (e.g., National Institute of Standards and Technology [NIST] and Wiley mass spectra library). This matching process is primarily based on a weighted dot product (or cosine similarity) between the query spectrum (unknown) and the library spectrum (reference) (Stein and Scott 1994). The compounds detected by GC–MS tend to be less polar and have lower molecular weights, such as benzene derivatives and polycyclic aromatic hydrocarbons (Adly et al. 2025; Lin et al. 2017). However, high‐molecular‐weight compounds can still be effectively analyzed by GC–MS when employing elevated column temperatures. For instance, Yang et al. (2016) successfully investigated the migration behavior of Irgafos 168 and tris(2,4‐di‐tert‐butylphenyl)phosphate using GC–MS with an optimized column temperature of 300°C, demonstrating the feasibility of this approach for thermally stable, low‐ or semivolatile additives in FCMs.

**FIGURE 1:**
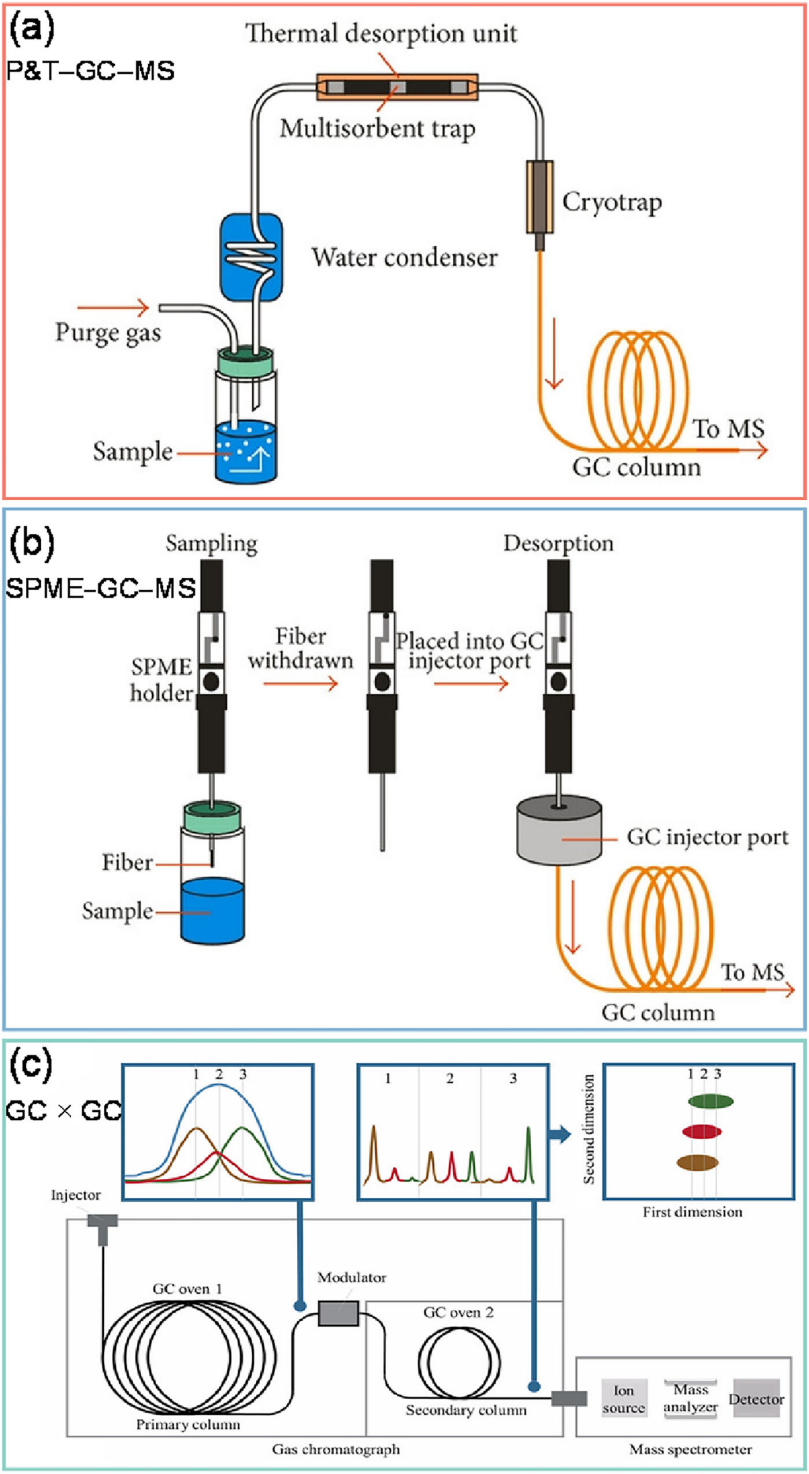
Advanced sample preparation and multidimensional separation strategies for food packaging migrants. (a) Purge and trap coupled to gas chromatography and mass spectrometry (P&T–GC–MS). (b) Solid‐phase microextraction coupled to GC–MS (SPME–GC–MS). (b) Schematic diagram of comprehensive two‐dimensional gas chromatography (GC × GC) illustrating the modulation process that enhances peak capacity, enabling the effective separation of complex mixtures. (a, b) Reprinted from Schmidt and Podmore (2015) with permission from Hindawi (licensed under CC‐BY 3.0, https://creativecommons.org/licenses/by/3.0/). (c) Reprinted from Chien et al. (2023) with permission from Wiley.

In migration testing of food packaging, standardized food simulants (e.g., 3% acetic acid, 10% ethanol, and 20% ethanol) are typically employed as substitutes for real food matrices to evaluate the leaching of chemical substances. Therefore, sample pretreatment methods are required to isolate and concentrate the migrated analytes into GC‐compatible solvents prior to instrumental analysis. In addition to the traditional extraction methods, such as solid‐phase extraction (SPE) (Fasano et al. 2012) and liquid–liquid extraction (LLE) (Onghena et al. 2014), solvent‐free sampling techniques, including static headspace (SHS), dynamic headspace (e.g., purge and trap [P&T]), and solid‐phase microextraction (SPME), have garnered increasing attention in FCC analysis. SHS is widely recognized for its robustness and simplicity in characterizing major volatile migrants. However, since it involves the injection of only a fraction of the gas phase at equilibrium without an inherent enrichment step, SHS exhibits relatively lower sensitivity compared to P&T and SPME (Lestido‐Cardama et al. 2021; Nerin et al. 2009). In contrast, P&T offers superior sensitivity by continuously purging and trapping analytes onto a sorbent interface, as shown in Figure 1a, making it particularly effective for the enrichment of trace‐level volatile migrants. Consequently, several studies have leveraged the high sensitivity of P&T to characterize volatile chemicals in food packaging materials and internal can coatings (López Sanvicente et al. 2025; Lestido‐Cardama et al. 2021; Vazquez‐Loureiro, Cariou, et al. 2025; Vazquez‐Loureiro, Cotos Suárez, et al. 2025). For instance, Vazquez‐Loureiro, Cariou, et al. (2025) utilized P&T–GC–MS to characterize volatile compounds in food contact bioplastics. Owing to the exceptional sensitivity of this technique, a total of 125 volatile compounds were detected across various biobased and biodegradable materials. Of these, 100 substances were tentatively identified, ranging from monomers and starting materials to additives, solvent residues, and degradation products, thereby providing a comprehensive chemical profile of volatile migrants in biobased and biodegradable packaging materials.

SPME, a highly integrated technique consolidating sampling, extraction, and pre‐concentration (Figure 1b), is also widely adopted for analyzing migrants from food packaging materials (Asensio et al. 2020; Estremera et al. 2025; Song et al. 2019; Su, Vera, Nerin, et al. 2021; Xu et al. 2016). Unlike P&T, which utilizes sorbent traps for the exhaustive collection of volatiles, SPME relies on a stationary‐phase‐coated fiber for the targeted enrichment of analytes from either the headspace or the liquid phase. The extraction efficiency is primarily governed by the partition coefficient between the fiber coating and the sample matrix, imparting an inherent selectivity to the method (Prosen and Zupančič‐Kralj 1999). Similar to P&T, SPME also offers high analytical sensitivity. Su et al. (2020) demonstrated that direct immersion SPME–GC–MS could achieve a low limit of detection (LOD) of 0.1 ng/g for FCM‐related compounds. Nevertheless, the careful selection of an SPME fiber coating is essential to accommodate the diverse physicochemical properties of various migrants.

### 3.2 Comprehensive Two‐Dimensional (2D) Gas Chromatography

When analyzing complex migrant mixtures, conventional 1D GC–MS systems often suffer from chromatographic co‐elution, a phenomenon where multiple analytes with similar physicochemical properties elute from the column simultaneously and overlap within a single peak. This overlap restricts the system's capacity to resolve closely related compounds, frequently resulting in complex mass spectra that hinder confident structural elucidation. To overcome this limitation, comprehensive 2D gas chromatography (GC × GC) has emerged as a powerful analytical technique for the characterization of FCCs. Figure 1c shows the schematic diagram of the GC × GC technique. The primary advantage of GC × GC is the vast improvement in chromatographic resolution over conventional 1D GC (Biedermann and Grob 2019; Li, Chen, et al. 2023). In the GC × GC plot, the compounds co‐eluting from the first‐dimension column can be well‐resolved along the second dimension (Biedermann and Grob 2019). Hao et al. (2022) show that two peaks (2,4‐di‐tert‐butylphenol and dimethyl isophthalate) co‐eluted on the DB‐5MS column can be fully separated by the DB‐17MS column. The increased peak capacity provided by GC × GC enables more peaks to be resolved and subsequently unambiguously identified, especially for the previously co‐eluting compounds. For example, the work of Adahchour et al. (2003) unequivocally identified methional in the second‐dimension chromatogram, after the elimination of the predominant interference from 2‐heptanone. Li et al. (2022) combined a semi‐nonpolar HP‐5MS capillary column with a semi‐polar DB‐17 capillary column, and successfully identified 1247 volatile chemicals in polyethylene terephthalate (PET) packaging materials.

Beyond the enhanced peak capacity, another advantage of GC × GC is the systematic ordering of structurally related compounds on the 2D chromatogram according to their physicochemical properties (e.g., volatility, polarity). Such an ordered arrangement is highly beneficial as it resolves homologous series (e.g., alkanes, aromatics, oligomers) and functional groups into distinct, recognizable clusters, significantly accelerating pattern identification (Biedermann and Grob 2019). The work of Biedermann and Grob (2019) shows a comprehensive GC × GC plot of a hexane/ethanol extract from recycled paperboard FCMs. Notably, compounds differing by a repeating chemical unit, such as *n*‐alkanes, form approximately horizontal rows with regular intervals between signals. The solid line in the plot connects the centers of the *n*‐alkane signals, while a broken line just below it indicates a series of *n*‐alkyl cyclopentanes and *n*‐alkyl cyclohexanes. This clear regularity allows researchers, upon the identification of just a few key compounds, to systematically deduce the likely chemical structures of other signals across the chromatogram. This faculty significantly streamlines the identification of structurally related compounds in FCMs, such as oligomers and degradation products with the same structural elements.

While GC × GC significantly improves the resolution and identification of FCCs via 2D separation, its application is constrained by notable technical and practical limitations. The first limitation is the intricate method development. Setting up a GC × GC method is significantly more complex than 1D GC. Analysts must optimize not only the two columns (phase selection, dimensions, flow) and oven temperature program but also the modulator settings (modulation period, hot/cold jet timings, or flow rates). The interdependency of these parameters makes method development tedious and often requires specialized expertise. Furthermore, GC × GC generates massive, high‐dimensional data files, creating a substantial workload for processing and storage, which mandates the use of specialized software and high‐performance computing resources (Mondello et al. 2025). These practical disadvantages may impede the broader implementation of the GC × GC technique for the routine analysis of food packaging migrants.

### 3.3 GC Coupled to HRMS (GC–HRMS)

While low‐resolution GC–MS systems remain the most prevalent and widely employed mass spectrometers for analyzing volatile and semivolatile migrants from food packaging materials, recent advances in HRMS have expanded analytical capabilities in this field. In recent years, commercially available GC–HRMS platforms, equipped with time‐of‐flight (TOF) or Orbitrap mass analyzers, have emerged as powerful alternatives. Notably, several of these high‐resolution systems have already demonstrated their applicability in food packaging research, offering enhanced mass accuracy and improved compound identification compared to conventional low‐resolution instruments (Li, Chen, et al. 2023; Miralles et al. 2021; Sapozhnikova 2021; Sapozhnikova and Stroski 2025).

The ability to provide accurate mass measurements, typically below 2 ppm for Orbitrap and 5 ppm for TOF analyzers (Liu et al. 2019), seems the most compelling feature of GC–HRMS instrumentation. The high mass accuracy of GC–HRMS enables the elimination of background interferences and isobaric fragments during spectral deconvolution, allowing for the acquisition of cleaner mass spectra. This capability significantly contributes to the correct identification of analytes. Despite these significant technological advancements, the fundamental workflow for data analysis remains largely unchanged. The identification of migrated compounds by GC–HRMS is primarily achieved through spectral library matching, typically employing reference databases such as the NIST library or other commercial spectral collections. For example, Li, Zeng, et al. (2023) successfully identified 344 volatile organic compounds (VOCs) in food‐contact paperboard through GC coupled to quadrupole time‐of‐flight mass spectrometry (GC–QTOF), employing identification criteria of NIST mass spectral match factor >800 and absolute retention index (RI) difference <30 units. Therefore, the utilization of GC–HRMS for the identification of food packaging migrants has introduced a fundamental conflict: while GC–HRMS instruments can acquire mass spectra with high mass accuracy, the established reference libraries, such as NIST, are composed almost entirely of low‐resolution MS spectra. To fully exploit the potential of GC–HRMS in food packaging studies, it is essential to integrate high‐resolution accurate mass (HRAM) data with traditional spectral matching.

**FIGURE 2:**
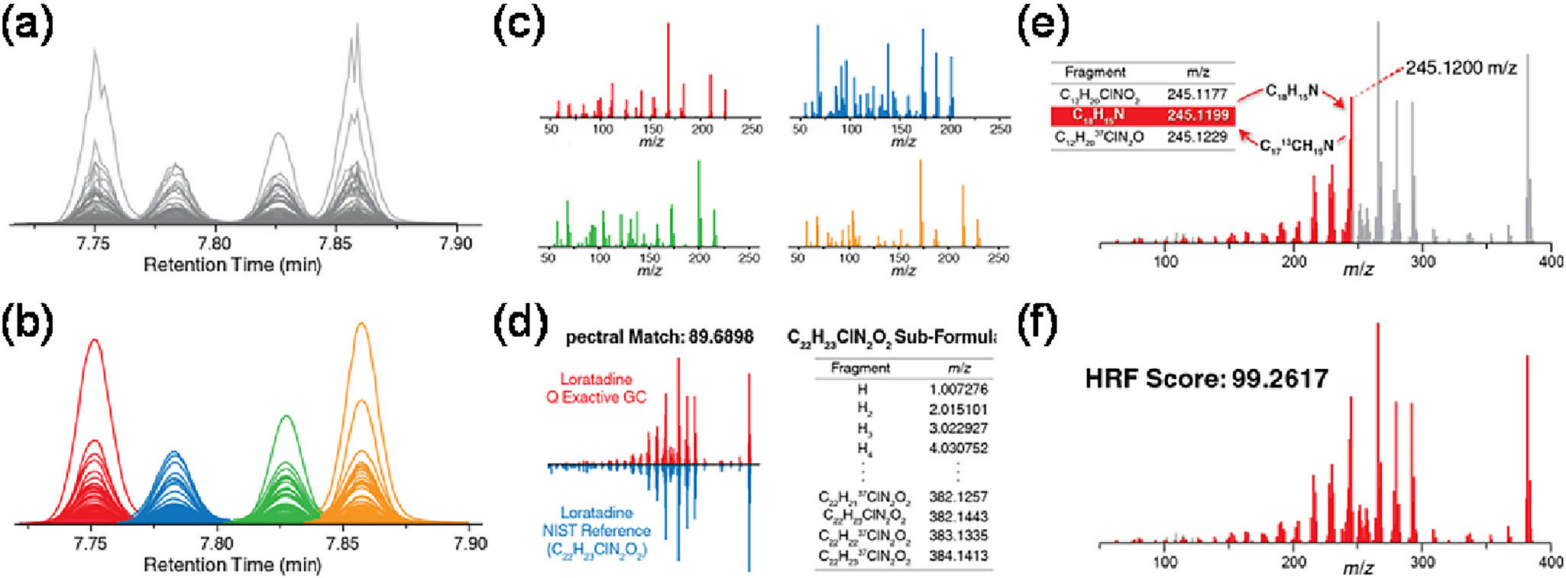
Systematic high‐resolution filtering (HRF) workflow for enhancing identification confidence in gas chromatography coupled to high‐resolution mass spectrometry (GC–HRMS). The HRF strategy leverages high‐resolution accurate mass (HRAM) data to validate candidate matches from traditional low‐resolution libraries. (a) Peaks observed across consecutive scans are condensed into data features. (b) Features are smoothed and grouped based on elution apex. All features within a group are assumed to arise from a singular precursor. (c) Individual spectra are derived from feature groups (using average *m*/*z* and apex intensity) and can then be submitted for spectral matching. (d) A strong spectral match of an experimentally derived spectrum of loratadine against the corresponding NIST reference spectrum. All subformulas from C_22_H_23_ClN_2_O_2_ are generated and sorted by exact formula mass less an electron. (e) Subformulas are matched to peaks in ascending order based on *m*/*z*. For each matched theoretical fragment, a variant containing appropriate heavy isotopes is created and placed into the list of subformulas in sorted order. (f) For the high‐resolution spectrum of loratadine, 99.2617% of the measured ion current can be annotated with a subformula of C_22_H_23_ClN_2_O_2_. Reprinted from Kwiecien et al. (2015) with permission from the American Chemical Society.

To fully exploit the dual advantages of GC–HRMS accurate mass measurements and conventional extensive spectral libraries, a novel filtering strategy has been proposed: high‐resolution filtering (HRF) (Kwiecien et al. 2015). The basic principle of HRF is based on a fundamental tenet of mass spectrometry: in the EI mass spectrum of a pure compound, every fragment ion must be composed of a subset of the atoms from the parent molecule. HRF leverages this principle to assess the plausibility of candidate identifications returned by traditional library searches (Kwiecien et al. 2015). A schematic representation of the HRF workflow is provided in Figure 2. Generally, HRF begins with spectral deconvolution of the raw GC–HRMS data to extract high‐resolution mass spectra. These spectra are then converted to a pseudo‐unit resolution format for a traditional search against libraries like NIST, yielding a list of candidate identifications ranked by spectral similarity. For each high‐scoring candidate, the algorithm computationally generates a comprehensive list of all possible subformulas, that is, unique atomic combinations that are subsets of the parent molecule's chemical formula. The pivotal step involves returning to the original high‐resolution experimental spectrum and attempting to annotate every fragment ion using this list of theoretical subformulas within a narrow mass tolerance (e.g., mass tolerance of ±10 ppm). The HRF score is then calculated as the percentage of the total measured ion current that can be successfully explained by these subformulas. A high HRF score indicates that the candidate's formula is chemically plausible and can account for the observed fragmentation pattern, thus lending high confidence to the identification. Currently, several studies have incorporated the HRF score in the identification of food packaging migrants (Miralles et al. 2021; Sapozhnikova 2021; Sapozhnikova et al. 2021). For example, Sapozhnikova (2021) identified 35 migrated chemicals in paper‐based food packaging using identification criteria of NIST MS Library match score >90%, HRF score >80%, mass accuracy <5 ppm, and RI difference <50 units. The combination of HRF and spectral match score, as well as other identification evidence, effectively reduces false‐positive identifications.

### 3.4 Combination Between EI and Soft Ionization Techniques

Although GC–HRMS is increasingly employed for analyzing migrated chemicals from food packaging, it should be noted that most current GC–HRMS instruments operate with EI at 70 eV in scan mode. The “hard” ionization characteristic of EI presents a significant trade‐off. Although it reliably generates stable and reproducible fragmentation patterns for library matching, the high energy of the process frequently leads to the complete fragmentation of many compounds, resulting in the absence of the molecular ion (M^+•^) in the mass spectrum. For example, in the work of Sapozhnikova (2021), molecular ions were detected for only 10 out of the 35 migrated compounds identified using GC coupled to quadrupole Orbitrap mass spectrometry (GC–Q‐Orbitrap). Consequently, in cases where a compound is absent from standard spectral libraries, or when an excessive number of candidates are proposed by library matching, the absence of the molecular ion makes the determination of a molecular formula and the subsequent structural elucidation a challenging task.

**FIGURE 3:**
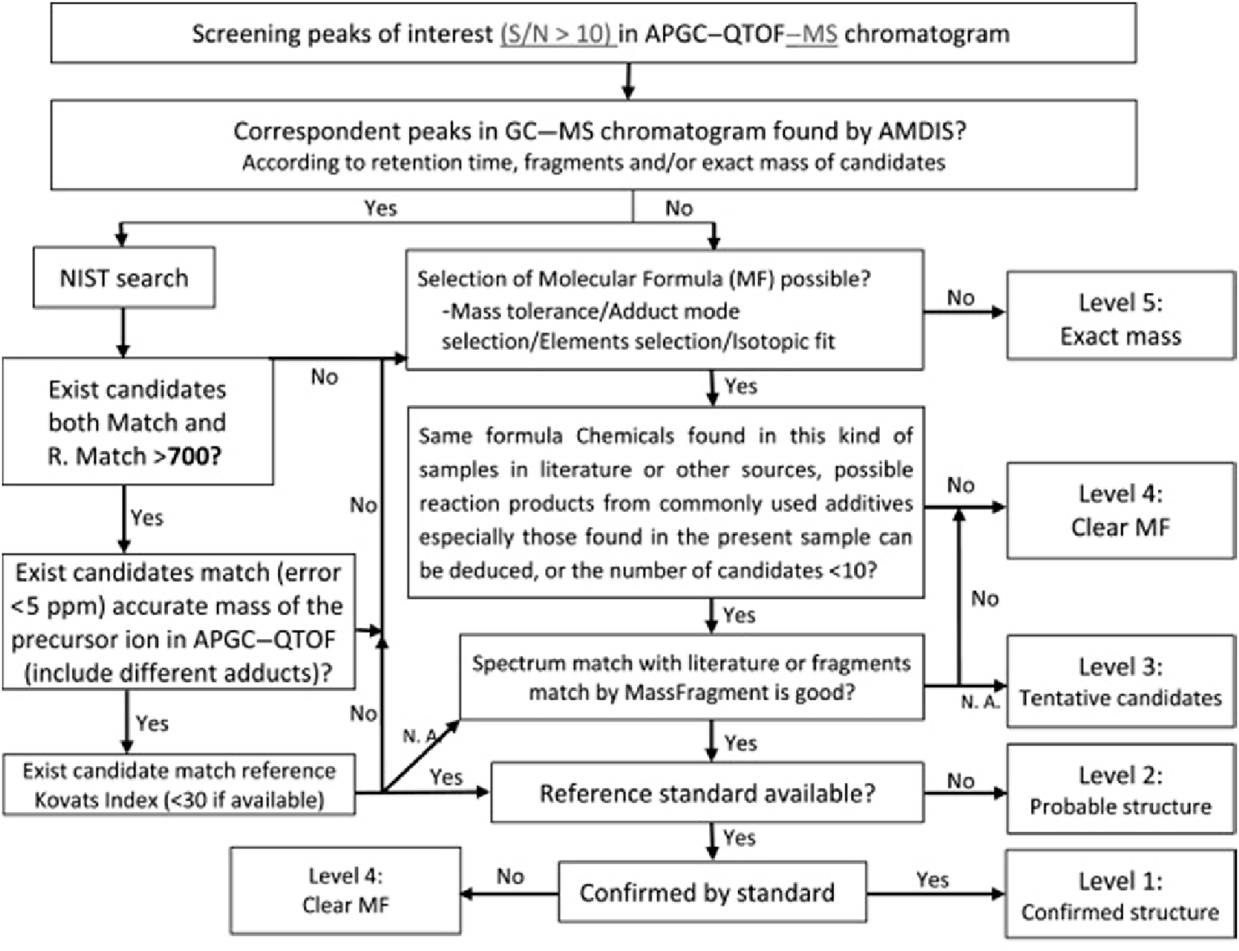
Integrated nontargeted analysis (NTA) workflow for unidentified migrants. The flowchart depicts the synergistic use of gas chromatography–atmospheric pressure chemical ionization coupled to quadrupole time‐of‐flight mass spectrometry (GC–APCI–QTOF) for accurate mass and molecular formula determination, and gas chromatography coupled to mass spectrometry (GC–MS) for NIST spectral library matching, a combined approach designed to increase the identification confidence of unknown non‐intentionally added substances (NIAS) in food packaging materials. Reprinted from Su et al. (2019) with permission from Elsevier.

To address this challenge, several researchers have combined EI with soft ionization techniques (e.g., atmospheric pressure chemical ionization [APCI]) for the analysis of chemicals migrating from food packaging. This hybrid approach aims to simultaneously acquire both the characteristic fragment ion mass spectra from EI and the molecular ion information from the soft ionization source (Canellas et al. 2012, 2014; Osorio et al. 2019; Su et al. 2019). Figure 3 illustrates the workflow for identifying volatile and semivolatile compounds in food packaging by combining GC–APCI–QTOF and GC–EI–MS. Specifically, for an unknown peak, the initial step involves searching its GC–EI–MS spectrum against commercial libraries, such as NIST, to generate a list of candidate compounds. Subsequently, the high‐resolution accurate mass data provided by GC–HRMS equipped with a soft ionization source are utilized to validate the molecular formulas of these candidates. This approach enables the rapid elimination of numerous false positives, thereby greatly enhancing both the efficiency and accuracy of the identification process. For instance, in Su et al. (2019), a peak observed at 19.24 min in the GC–MS chromatogram yielded an ambiguous result upon a NIST library search of its EI spectrum, returning over 30 candidates with high match scores (>850). To resolve this ambiguity, the high‐resolution data from GC–APCI–QTOF were employed. The corresponding peak at 19.37 min provided a precursor ion with an accurate mass at mass‐to‐charge ratio (*m*/*z*) of 279.1589. This accurate mass was used as a filter to verify the molecular formulas of the initial candidates, successfully reducing the list from over 30 possibilities to only four structural isomers (diisobutyl phthalate, 1‐butyl 2‐isobutyl phthalate, dibutyl phthalate, and di‐sec‐butyl phthalate). The identity of this peak was subsequently confirmed by the injection of a diisobutyl phthalate reference standard. This synergistic analytical strategy effectively integrates the strengths of GC–MS and GC–HRMS, providing a more comprehensive, reliable, and efficient solution for the NTA of (semi‐)volatile compounds in food packaging.

## 4 LC–MS Utilized in the Analysis of IAS and NIAS

### 4.1 LC Coupled to HRMS (LC–HRMS)

While GC–MS excels in analyzing volatile and semivolatile food packaging migrants, its limitations in analyzing thermally labile, highly polar, or high‐molecular‐weight compounds (e.g., certain antioxidants, oligomers) are evident. In recent years, LC–HRMS has emerged as a powerful alternative, leveraging its mild ionization process, superior mass accuracy, and dynamic range (Xian et al. 2012). This technique is particularly transformative for NTA and trace‐level detection of polar and high‐molecular‐weight compounds in food packaging (Alberto Lopes and Tsochatzis 2023; Nerin et al. 2022; Sanchis et al. 2017; Wrona and Nerin 2020). QTOF and Orbitrap are the two predominant types of mass analyzers employed for LC–HRMS in the field of food packaging analysis. In a 2019 review by Martínez‐Bueno et al. (2019) concerning migrants from plastic food packaging, it was noted that QTOF systems were more frequently utilized than Orbitrap instruments in this field. This disparity was attributed in part to the higher cost of Orbitrap instrumentation and its more recent introduction to the mass spectrometry market. Compared to QTOF analyzers, Orbitrap instruments are capable of providing more accurate mass measurements (Krauss et al. 2010; Wang and Li 2022). For instance, the Q Exactive platform can achieve a resolving power as high as 140,000 full width at half maximum (FWHM) at *m/z* 200, with mass errors often below 1 ppm (Yan et al. 2022). Resolving power is defined as the ratio of the *m/z* of an ion to its peak width at half of the maximum peak height (Murray 2022). A high resolving power can facilitate the effective separation of isobaric compounds. By successfully resolving these closely spaced peaks, the system ensures the high mass accuracy necessary for the unambiguous assignment of molecular formulas to unknown migrants in complex food packaging matrices. However, it should be noted that the high resolving power of Orbitrap analyzers involves a trade‐off with acquisition speed. A slower scan rate may yield an insufficient number of data points to accurately define a chromatographic peak, potentially leading to false negatives in food packaging analysis (Stincone et al. 2023). This phenomenon is particularly pronounced for low‐abundance peaks resulting from trace concentrations or poor ionization efficiency of analytes. Furthermore, the high‐efficiency columns with smaller particle sizes generate narrower peaks, which impose even more stringent requirements on scan rate to ensure adequate sampling and peak integrity (Defossez et al. 2023; Huang et al. 2021).

### 4.2 LC–Ion Mobility Spectrometry–HRMS (LC–IMS–HRMS)

The origin of IMS dates back to the pioneering work of Thomson and Rutherford in 1896, who investigated the mobility of ions in various gases (Thomson and Rutherford 1896). The fundamental principle of IMS is the separation of ions based on their differential migration through an inert buffer gas under the influence of an electric field (Dodds and Baker 2019). The combination of IMS with LC–HRMS creates a powerful analytical platform. The complementary separation provided by these two analytical methods enables the high‐dimensional analysis of analytes in complex systems. Notably, the IMS separation process occurs on a millisecond timescale (D'Atri et al. 2018), and this rapid process makes it seamlessly compatible with conventional LC–HRMS analytical workflows. The collision cross section (CCS) derived from IMS is a stable and intrinsic physicochemical property of an ion, reflecting its size, shape, and charge distribution. Technically, the CCS value is determined by measuring the drift time of an ion as it travels through a drift tube; the drift time is inversely proportional to the ion's mobility, which is subsequently converted into a CCS value by via the Mason–Schamp equation accounting for environmental factors such as gas temperature, pressure, and the reduced mass of the ion and buffer gas (Dodds and Baker 2019). These characteristics make CCS a highly reliable parameter that can be used as additional evidence of identification for analytes (Dodds et al. 2020; Mullin et al. 2020; Regueiro et al. 2016). Applications of LC–IMS–HRMS are now expanding into the field of food packaging analysis. For instance, Song and coworkers constructed the first CCS database for 675 extractables and leachables (E&L) from FCMs (Song, Canellas, et al. 2022a). In a related study, Vera et al. (2024) utilized LC–IMS–QTOF to assess the migration of per‐ and polyfluoroalkyl substances (PFAS) from various food packaging materials.

A significant benefit of LC–IMS–QTOF is its ability to enhance the quality of mass spectra acquired in data‐independent acquisition (DIA) mode (Celma et al. 2021; Celma et al. 2020; Regueiro et al. 2016). In most current LC–IMS–QTOF systems, the ion mobility cell is positioned between the ion source and the collision cell. This configuration ensures that a precursor ion and its corresponding product ions share the same drift time. By aligning precursor and fragment ions based on both retention time (RT) and drift time, many interfering ions from co‐eluting compounds and background noise can be effectively eliminated (Celma et al. 2020; Song et al. 2023). This process simplifies the resulting mass spectra and facilitates subsequent spectral interpretation. The spectral cleanup effect of IMS has also been demonstrated in the analysis of migrants from food packaging. For example, Canellas et al. (2020) compared the high‐energy mass spectra of 1‐tetradecanesulfonic acid acquired with and without drift time alignment. This comparison revealed that aligning the data by drift time effectively removed a large number of interfering ions and produced a cleaner mass spectrum.

### 4.3 Data Acquisition Method

**FIGURE 4:**
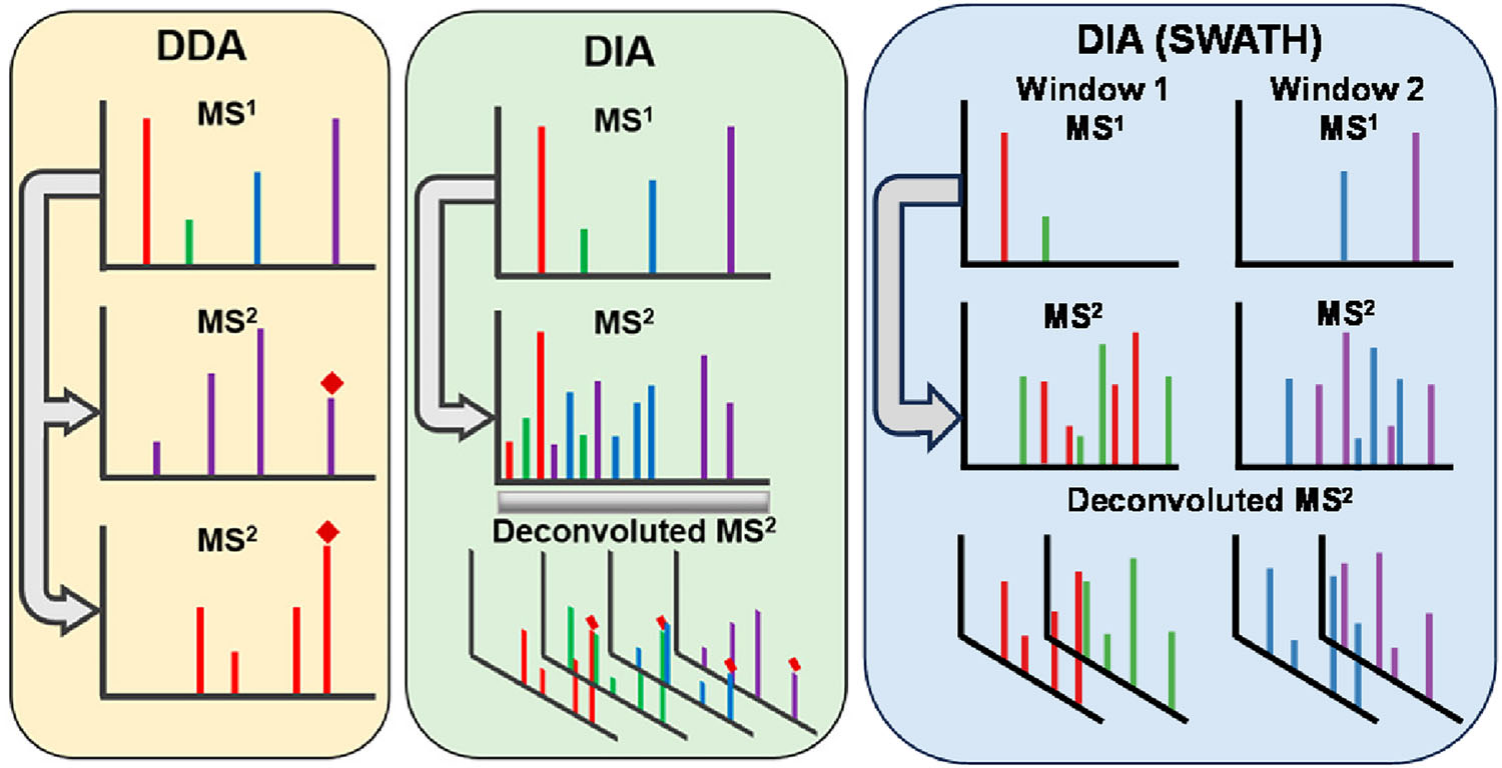
Comparison of data acquisition strategies in high‐resolution mass spectrometry. Data‐dependent acquisition (DDA) selectively fragments only the most intense precursor ions based on predefined thresholds, which may result in the omission of trace‐level analytes. Traditional data‐independent analysis (DIA) typically uses broad windows for simultaneous fragmentation, whereas sequential window acquisition of all theoretical fragment ion spectra (SWATH) improves selectivity by fragmenting the ions in narrower, sequential windows. The schematic diagram of DDA and DIA was reprinted from Guo and Huan (2020) with permission from the American Chemical Society; the schematic diagram of SWATH was created with PowerPoint.

In the analysis of food packaging migrants by LC–HRMS, data‐dependent acquisition (DDA) (Bhattarai et al. 2024; Sapozhnikova and Nunez 2022; Wang et al. 2020; Wu et al. 2024) and DIA (Aznar et al. 2012; Tisler and Christensen 2022; Vera et al. 2018) are two commonly used data acquisition methods. The schematic diagrams of DDA and DIA are shown in Figure 4.

#### 4.3.1 DDA

DDA, also known as information‐dependent acquisition (IDA), involves a core workflow with an initial full scan (MS^1^), from which the instrument automatically selects a limited number of precursor ions based on predefined criteria, such as intensity. These selected ions are then sequentially subjected to fragmentation to acquire their MS/MS structural information (Yang et al. 2023). The primary advantage of DDA is that it generates clean MS/MS spectra with minimal interference from co‐eluting compounds, making them highly amenable to subsequent library matching and structural elucidation. However, this selectivity also introduces inherent limitations, most notably its limited coverage. A significant number of compounds in low abundance that do not meet the predefined triggering criteria are not selected for fragmentation, and thus their MS/MS information is not acquired (Guo and Huan 2020). To enhance the acquisition range and data quality of MS/MS spectra for low‐abundance migrated chemicals, Taylor and Sapozhnikova (2022) employed a two‐stage data acquisition strategy. Initially, a preliminary matching of precursor ions from the full‐scan data was performed against the ChemSpider and E&L databases. Subsequently, the successfully matched precursor ions were added to a data‐dependent MS/MS (ddMS^2^) inclusion list and prioritized for fragmentation. This analytical strategy enabled the successful identification of 63 and 27 food packaging migrants in electrospray ionization (ESI) positive and negative modes, respectively. While this prioritized approach effectively captures MS/MS spectra for trace‐level components, its overall identification efficacy is heavily reliant on the comprehensiveness of existing databases. Consequently, the identification of truly unknown compounds remains challenging, as the strategy exclusively targets precursor ions with prior database matches.

#### 4.3.2 DIA

In contrast to the selective nature of DDA, DIA employs a comprehensive fragmentation strategy. The core concept is the systematic and unbiased fragmentation of all precursor ions within predefined *m/z* windows, thereby acquiring a complete record of MS/MS fragment information for all compounds in a sample (Fröhlich et al. 2024). The primary advantage of DIA lies in this unparalleled coverage, which theoretically ensures that no compound's MS/MS information is missed. However, its main disadvantage is that the correspondence between precursor and fragment ions is lost, and the resulting MS/MS spectra are highly complex, posing a significant challenge for subsequent spectral deconvolution (Guo and Huan 2020). Vera et al. (2018) employed ultraperformance liquid chromatography coupled to QTOF operated in MS^E^ mode (UPLC–QTOF–MS^E^) to identify chemicals migrating from polypropylene (PP) food packaging. As a conventional DIA method, MS^E^ operates by alternating between low‐ and high‐collision‐energy scans. This approach enables the simultaneous acquisition of both precursor ion information and fragmentation data within a single chromatographic run. The high‐energy scan employs a collision energy ramp to systematically acquire fragment ion spectra across varying energy levels. This approach ensures the generation of rich structural information for diverse chemical classes, with typical ramp settings ranging, for instance, from 15 to 40 V in food packaging analysis (Vera et al. 2018).

In recent years, the evolution of mass spectrometers toward faster acquisition times and higher duty cycles has facilitated the emergence of a novel DIA method: sequential window acquisition of all theoretical fragment ion spectra (SWATH) (Gillet et al. 2012). SWATH offers a compelling balance between comprehensive coverage and high MS/MS data quality. The technique operates by partitioning the full mass range into numerous consecutive and independent narrow precursor isolation windows. Within each of these windows, the instrument systematically and cyclically fragments all ions (Figure 4). Unlike DDA, which typically prioritizes high‐abundance precursors and may overlook trace‐level components, SWATH ensures a more comprehensive and unbiased data acquisition of the entire sample. Furthermore, because the windows are relatively narrow, the complexity of the resulting MS/MS spectra is significantly reduced compared to traditional wide‐window DIA (Zhu et al. 2014). Consequently, the correlation between precursor and fragment ions is far stronger, establishing a robust foundation for subsequent accurate qualitative analysis. Several recent studies have employed the SWATH technique for the analysis of food packaging migrants (Bauer et al. 2019; Gomez Ramos et al. 2019; Tang et al. 2023). In one of these works, the data acquisition was performed by setting 10 first quadrupole isolation windows, which spanned a mass range from *m/z* 100 to 950. The use of these relatively narrow windows significantly improved the quality of the resulting MS/MS spectra, allowing for the identification of 26 migrants from multilayer plastic packaging (Gomez Ramos et al. 2019).

#### 4.3.3 Combination Between DDA and DIA

To leverage the respective advantages of DDA and DIA in the field of food packaging, several researchers have developed hybrid strategies (Chen et al. 2024; Wang, Xiao, et al. 2022; Xiao et al. 2023). For instance, Wang, Xiao, et al. (2022) implemented a nontargeted screening method based on characteristic fragments to identify OPEs in FCMs. Their approach utilized DIA for an initial, comprehensive screening of characteristic fragment ions, followed by a targeted DDA experiment to acquire high‐quality MS/MS for the corresponding precursor ions. This combination of DIA and DDA ensures exhaustive coverage during the initial fragment ions screening while yielding clean, high‐confidence mass spectra for identification, thereby overcoming the limitations of using either mode in isolation. Consequently, a total of seven novel OPEs were unambiguously identified in food packaging through this hybrid strategy (Wang, Xiao, et al. 2022).

## 5 Data Processing Strategies Used in the Identification of Food Packaging Migrants

After the acquisition of massive data generated from MS, researchers have established a multitiered data processing strategy for the identification of migrating substances. Depending on the a priori knowledge of the analytes and the availability of the reference standards, current processing methods can be broadly classified into three categories: targeted analysis, suspect screening, and NTA. The systematic workflows of the three data processing strategies have been illustrated in Krauss et al. (2010), each representing a distinct trade‐off between target specificity and data processing complexity.

Targeted analysis, which focuses on compounds with known structures and available reference standards, is a foundational method in the analysis of migrants from food packaging materials. However, this approach is inherently limited by the availability of standards, a challenge that has persisted for plastic FCMs like polyolefins and PET for decades (Bradley and Coulier 2007; Schreier et al. 2023). The recent widespread use of novel food packaging materials and additives has further exacerbated this issue by introducing numerous potential emerging contaminants for which no standards exist, making comprehensive coverage by targeted analysis alone unfeasible. To address this gap, researchers have developed a suspect screening and NTA strategy that enables the tentative identification of migrants (Liagkouridis et al. 2025). This suspect screening strategy relies on suspect lists that contain, at a minimum, molecular formulas, but may also be augmented with characteristic ion information and predicted RT and CCS values. Furthermore, when the analytes are entirely unknown and lack any reference information, NTA is required. This approach mainly utilizes HRMS to infer the molecular structures based on the precursor and fragment ions, as well as the prior knowledge about the food packaging materials and the expert experience in this field. The detailed applications of these three strategies in the analysis of food packaging migrants will be discussed below.

### 5.1 Targeted Analysis

Targeted analysis represents one of the most common and extensively applied analytical strategies in food packaging studies. This approach primarily involves the identification and quantification of migrated chemicals using reference standards. Compared to suspect screening and NTA, targeted analysis offers the advantages of high confidence in identifications. Table 2 presents several cases of targeted analysis in the field of food packaging, which cover a diverse range of targeted compounds, food packaging materials, and analytical techniques.

**TABLE 2:** Targeted analysis of migrants from food packaging.

Compounds identified	Food packaging materials	Food/food simulants	Analytical techniques	Reference
Eleven phthalates	Adhesive materials (labels and tapes)	Apples, avocados, and celery	GC–QqQ	Hou et al. 2021
Ten photoinitiators, eight phthalates and non‐phthalate plasticizers	Paper and cardboard food packaging	Food simulants (50% and 95% EtOH and Tenax) and foodstuffs (rice, cereals, and milk powder	UPLC–Q‐Orbitrap	Blanco‐Zubiaguirre et al. 2021
Ten synthetic phenolic antioxidants	116 food plastic packaging materials	10%, 50%, and 90% ethanol	UPLC–QqQ	Yin et al. 2025
Thirteen organophosphate esters	Polypropylene films	Full‐fat milk powder	UPLC–QqQ	Miao et al. 2024
Twenty bisphenol analogues	Polystyrene take‐out containers	Tap water, 10% and 50% ethanol, corn oil, steamed rice	UPLC–QqQ	Zhao et al. 2023
Bisphenol S and four other color developers	Food thermal labels	Fish and 95% ethanol	UPLC–QTOF	Xu, Tian, et al. 2023
Fourteen PFAS	Paper‐based food packaging and Teflon	Tenax	UPLC–IMS–QTOF	Vera et al. 2024
Six first series of PET oligomers (from dimer to heptamer)	PET food trays	50% ethanol	UPLC–QTOF	Colombo et al. 2024
Three organophosphite antioxidants and three organophosphate esters	Five types of food packaging	Methanol, 3% acetic acid, and 0.001 mol/L NaOH	UPLC–QqQ	Xing et al. 2023
Antioxidants and their degradation products	Food packaging materials comprised 18 types of resins	Water, 4% acetic acid, 20% and 95% ethanol, *n*‐heptane, and isooctane	UPLC–QqQ	Kim et al. 2023
Melamine and its derivatives	Bamboo‐based food contact materials	3% acetic acid	UPLC–QqQ	Vazquez‐Loureiro, Cotos Suarez, et al. 2025

Abbreviations: GC–QqQ, gas chromatography coupled to triple quadrupole; NaOH, sodium hydroxide; PET, polyethylene terephthalate; PFAS, per‐ and polyfluoroalkyl substances; UPLC–IMS–QTOF, UPLC coupled to ion mobility spectrometry and QTOF; UPLC–Q‐Orbitrap, ultra‐performance liquid chromatography coupled to quadrupole and Orbitrap mass spectrometry; UPLC–QqQ, UPLC coupled to QqQ; UPLC–QTOF, UPLC coupled to quadrupole time‐of‐flight mass spectrometry.

#### 5.1.1 Types of Chemicals Monitored by Targeted Analysis

Currently, targeted analysis primarily focuses on chemicals of high concern or those under regulatory scrutiny, such as phthalate esters (PAEs), bisphenols, photoinitiators, OPEs, and PFAS (Blanco‐Zubiaguirre et al. 2021; Li et al. 2024; Miao et al. 2024; Vera et al. 2024; Wang, Liu, et al. 2022; Zhao et al. 2023). Many of these FCCs are known to disrupt the human endocrine system, potentially leading to adverse health outcomes such as obesity and reproductive toxicity (He et al. 2025; Muncke et al. 2023; Zhang et al. 2017). More recently, the health effects of oligomers have garnered increasing attention from researchers. For instance, studies have shown that polylactic acid (PLA) oligomers can induce acute intestinal and colon inflammation (Wang et al. 2023). Similarly, recent research has demonstrated that PET oligomers, depending on their size and end‐group chemistry, can interact with deoxyribonucleic acid and trigger cellular immune responses (Djapovic et al. 2025). A critical issue in current risk assessment regarding oligomers is the conventional assumption that oligomers undergo complete hydrolysis into their respective monomers within the gastrointestinal tract, thereby suggesting that toxicological evaluation of the monomers alone is sufficient. However, empirical evidence suggests that this assumption is often not factual, particularly for cyclical oligomers, such as those found in PET and styrene, which exhibit high stability and persistence in biological systems (Alberto Lopes and Tsochatzis 2023; Diamantidou et al. 2022). To generate the necessary exposure data for oligomers, several research groups have begun to synthesize or isolate the oligomer standards from the corresponding raw polymers in order to quantify their migration levels (Alberto Lopes et al. 2021; Canellas, Vera, Song, et al. 2021; Diamantidou et al. 2023; Ruperez et al. 2024; Tsochatzis et al. 2020). In one such study, researchers isolated six PA6 oligomers and three PA66 oligomers and subsequently assessed their migration into various foods, including beans, chicken soup, whole milk, and sunflower oil (Canellas, Vera, Song, et al. 2021).

Besides oligomers, degradation products derived from additives (Remezov et al. 2025; Xing et al. 2023; Yang et al. 2016) represent another category of NIAS that is often subject to targeted analysis owing to potential health risks (Bou‐Maroun et al. 2023; Cui et al. 2022; Ren et al. 2023). Notably, antioxidants represent a primary focus within this category. Their extensive use as essential stabilizers, coupled with a high potential for migration, ensures significant levels of human exposure through food contact. A prominent example is Irgafos 168 and its degradation products. While Irgafos 168 itself is a common migrant, its breakdown products, particularly tris(2,4‐di‐tert‐butylphenyl)phosphate and 2,4‐di‐tert‐butylphenol, pose notable hazard properties. Recent toxicological studies have identified 2,4‐di‐tert‐butylphenol as an endocrine disruptor that promotes adipogenesis in human cells (Ren et al. 2023). Furthermore, due to its ubiquity and elevated concentrations frequently observed in migration tests, 2,4‐di‐tert‐butylphenol presents a substantial exposure risk to consumers (Han et al. 2024; Su, Vera, Nerin, et al. 2021). Similarly, tris(2,4‐di‐tert‐butylphenyl)phosphate was also identified as the predominant OPE contaminant in food packaging. For instance, the occurrence of three OPAs and their oxidized OPEs in plastic food packaging was investigated by targeted analysis. The study revealed that in all plastic packaging samples analyzed, the total concentration of OPEs (Σ_3_OPEs: 196–831 ng/g) was significantly higher than that of their OPA precursors (Σ_3_OPAs: <method quantification limits–124 ng/g). Among them, Irgafos 168 = O was the predominant OPE contaminant, while Irgafos 168 was the primary OPA (Xing et al. 2023).

#### 5.1.2 Low‐Resolution Mass Spectrometry (LRMS)‐ and HRMS‐Based Targeted Analysis

In targeted analysis studies, LRMS techniques, such as GC–MS, GC coupled to triple quadrupole (GC–QqQ), and UPLC–QqQ, have been predominantly utilized (Hou et al. 2021; Yang et al. 2016; Yin et al. 2025). In contrast, a smaller number of studies have employed HRMS platforms like UPLC–QTOF, UPLC–Q‐Orbitrap, and UPLC–IMS–QTOF (Blanco‐Zubiaguirre et al. 2021; Colombo et al. 2024; Vera et al. 2024). For targeted analysis, due to the availability of reference standards, the data provided by LRMS, specifically RT combined with two or three characteristic ions, are generally sufficient for the confident identification of target migrants. However, analyzing complex matrices, such as infant formula (characterized by high lipid and protein content), cereals, and rice, poses substantial challenges compared to food simulants (Blanco‐Zubiaguirre et al. 2021; Miao et al. 2024). These actual food matrices necessitate rigorous sample clean‐up procedures, such as lipid removal or protein precipitation, to effectively attenuate matrix effects and ensure data quality in MS analysis. Beyond matrix effects, interfering compounds from the sample matrix may co‐elute and share similar RT values and precursor–product ion pairs with the analytes of interest. This phenomenon can lead to false‐positive identifications and overestimated concentrations of the target compounds. The work of Pan and coworkers highlights a significant analytical interference in LRMS‐based targeted analysis (Pan et al. 2025). They found that γ‐carboxyethyl hydroxychroman (CEHC), a metabolite of vitamin E, co‐elutes and shares nearly identical ion pairs with perfluoropentanoic acid (PFPeA). This interference resulted in a substantial overestimation of the true PFPeA concentration, with reported levels being approximately 455 times higher than the actual value. HRMS‐based targeted analysis offers the advantage of high specificity in compound identification. The accurate mass measurements of both precursor ions and their resulting fragments effectively eliminate numerous background interferences. This capability provides a high degree of confidence for both the qualitative identification and subsequent quantification of analytes. Our own work has utilized multidimensional structural information, including RT, MS, MS/MS, and CCS values, to target PFAS in paper‐based food packaging materials. Their method demonstrated exceptional specificity, yielding highly confident identifications of the target PFAS compounds (Vera et al. 2024).

It should be noted that while the high mass accuracy of HRMS provides exceptional specificity, it often comes at the cost of a lower scan rate compared to LRMS. Consequently, in targeted analysis, HRMS generally exhibit lower sensitivity and higher LODs than LRMS (Pavon‐Perez et al. 2025; Stolker et al. 2004). However, a notable exception occurs in specific analytical scenarios. Certain migrated chemicals (i.e., phthalates) may preferentially form intense sodiated adducts [M + Na]^+^, rather than easily fragmented protonated molecules [M + H]^+^. These sodiated species are often resistant to fragmentation in the collision cell, which limits their efficient detection using the multiple reaction monitoring (MRM) mode on QqQ instruments (Kaufmann 2020). In such instances, HRMS‐based targeted analysis can achieve superior sensitivity compared to that of QqQ instruments by enabling the detection of the intact [M + Na]^+^ precursor ion. In summary, both LRMS‐ and HRMS‐based targeted analyses offer distinct advantages. The selection of an appropriate instrument for targeted analysis of food packaging migrants should consider sample complexity, the physicochemical characteristics of the analytes, and available experimental resources.

### 5.2 Suspect Screening

In contrast to targeted analysis, which relies on the direct comparison of a signal to that of an authentic reference standard, suspect screening enables the identification of a much broader range of known compounds. The core workflow of suspect screening involves matching features detected in a sample, typically pairs of RT and accurate *m/z*, against a predefined suspect list. This list can contain hundreds or even thousands of chemical candidates that are relevant to the sample, often compiled from scientific literature, chemical inventories, and specialized databases (Krauss et al. 2010). A successful match provides a tentative identification, which can then be prioritized for further confirmation. In recent years, several databases related to food packaging have been compiled, including the Chemicals associated with Plastic Packaging database (CPPdb) (Groh et al. 2019), the Food Contact Chemicals Database (FCCdb) (Groh et al. 2021), and the database on migrating and extractable FCCs (FCCmigex) (Geueke et al. 2023). The launch of these resources, as well as the increasing prevalence of HRMS, has significantly spurred the adoption of suspect screening for the analysis of food packaging materials. Table 3 summarizes several suspect screening studies in the food packaging field, covering diverse packaging materials, analytical techniques, and strategies for suspect list compilation.

**TABLE 3:** Suspect screening of migrants from food packaging.

Food packaging materials	Food simulants/Extraction	Analytical techniques	Suspect lists	Compounds identified	Reference
100 food contact materials	Ultrasonic extraction with hexane/acetone (v/v, 1:1)	UPLC–Q‐Orbitrap	124 OPEs compiled from literature	21 OPEs were identified	Wang, Xiao, et al. 2022
75 plastic food packaging materials	Ultrasonic extraction with hexane/acetone (v/v, 1:1)	UPLC–Q‐Orbitrap	A suspect list of 52 aryl di‐OPEs was established by deducting one ester group from aryl tri‐OPEs obtained from the literature	Five aryl di‐OPEs were identified	Xiao et al. 2023
39 drinking straws made from five types of materials	Ultrasonic extraction with methanol	UPLC–QTOF	Two suspect lists: PFAS Master List provided by the US EPA and literature	Two PFAS that were not included in the targeted analysis were identified	Boisacq et al. 2023
PA kitchenware	Dissolution and precipitation (hexafluoroisopropanol and methanol), migration (10% and 95% ethanol, 3% acetic acid)	UPLC–QTOF	Self‐build databases containing 682 plastic‐related chemicals	64 compounds were identified, includes PA oligomers, PEG oligomers, and IAS	Hu et al. 2021
PA spatulas	95% ethanol	UPLC–IMS–QTOF	Self‐build databases containing 675 compounds, and CPPdb and FCCdb	95 compounds were identified, including PA oligomers and IAS	Song, Canellas, et al. 2022b
Recycled HDPE	3% acetic acid and 95% ethanol	UPLC–QTOF	In‐house database, GC–MS‐identified chemicals, and CPPdb	83 compounds were identified	Su et al. 2023
Disposable biodegradable tableware	95% ethanol	UPLC–QTOF	In‐house database containing 1114 food packaging‐related compounds	303 chemical migrants were identified, including 123 oligomers	Chen et al. 2025
Reusable plastic bottles	Tap water	UPLC–QTOF	A compiled list containing 6130 plastic‐related compounds	42 compounds were identified	Tisler et al. 2024
Multilayer plastic pouch	10%, 20%, and 50% ethanol, 3% acetic acid	GC–Orbitrap, UPLC–QTOF	In‐house library containing 252 chemicals	26 chemicals were identified; most of the identified migrants are monomers and oligomers	Tang et al. 2023
Food contact paper and cardboard materials	Tenax	UPLC–Q‐Orbitrap	A home‐made database containing 2243 substances susceptible to migration and leaching	97 chemicals were identified	Blanco‐Zubiaguirre et al. 2020

Abbreviations: CPPdb, chemicals associated with plastic packaging database; FCCdb, food contact chemicals database; GC–MS, gas chromatography coupled to mass spectrometry; HDPE, high‐density polyethylene; IAS, intentionally added substances; OPEs, organophosphate esters; PA, polyamide; PEG, polyethylene glycol; PFAS, per‐ and polyfluoroalkyl substances; UPLC–IMS–QTOF, UPLC coupled to ion mobility spectrometry and QTOF; UPLC–Q‐Orbitrap, ultra‐performance liquid chromatography coupled to quadrupole and Orbitrap mass spectrometry; UPLC–QTOF, UPLC coupled to quadrupole time‐of‐flight mass spectrometry.

#### 5.2.1 Compilation of a Suspect List in Suspect Screening

The compilation of suspect lists for food packaging analysis is characterized by its high flexibility in both scale and scope. The number of compounds on such lists can range from tens to thousands (Jeon et al. 2025; Tisler et al. 2024; Xiao et al. 2023), depending on the research objective. These lists can be narrowly focused on a specific class of substances, such as OPEs (Wang, Xiao, et al. 2022), or they can be comprehensive, encompassing a wide variety of additives and NIAS (Bhattarai et al. 2025). Moreover, the level of detail within these lists can vary significantly. A basic list might contain only the molecular formula and monoisotopic mass for each entry (Hu et al. 2021). In contrast, more advanced lists are often enhanced with experimental or predicted data, such as RT and MS/MS fragmentation spectra (Song, Canellas, et al. 2022b), to increase the confidence of identification. A good application of suspect screening in food packaging analysis is the discovery of OPEs in FCMs (Wang, Xiao, et al. 2022). In this work, researchers began by constructing a suspect list of 124 OPEs compiled from previous studies; features detected in the samples were then matched against this list. For tentative identification, the experimental MS/MS spectra of these matched features were compared with either the spectra of reference standards or with MS/MS spectra reported in the literature. If a literature spectrum was unavailable, the fragmentation pattern was manually analyzed to assess its consistency with the suspected chemical structure. Using this strategy, 21 OPEs were successfully identified in the FCMs, 17 of which were further confirmed with reference standards (Wang, Xiao, et al. 2022).

**FIGURE 5:**
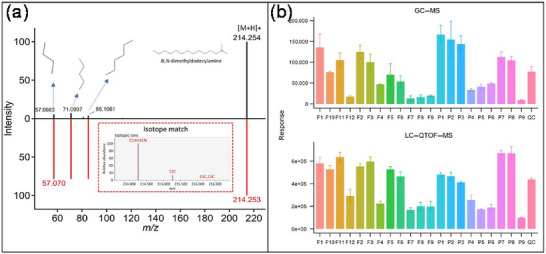
Identification of *N*,*N*‐dimethyldodecylamine. (a) Mirror plot comparison showing the alignment between the experimental MS/MS spectrum (upper, black) generated from liquid chromatography coupled to quadrupole time‐of‐flight mass spectrometry (LC–QTOF) and the in silico predicted fragmentation pattern (bottom, red) generated via MS‐FINDER. (b) Distribution profile of this compound across various food contact materials (FCMs) extracts, where distinct colors represent different samples analyzed by gas chromatography coupled to mass spectrometry (GC–MS) and LC–QTOF. Reprinted from Su et al. (2023) with permission from the American Chemical Society (licensed under CC‐BY 4.0, https://creativecommons.org/licenses/by/4.0/).

In addition to compiling suspect lists from published literature and chemical inventories, a distinctive approach has been proposed that utilizes identification results from a parallel GC–MS analysis of the same samples. In practice, compounds identified with high confidence in GC–MS, typically via robust EI library matching, are converted into a customized suspect list containing their molecular formulas and structures. This list is then imported into the LC–HRMS workflow, where the theoretical exact masses of corresponding adducts (e.g., [M + H]^+^ or [M + Na]^+^) are matched against the measured *m/z* values of features detected in the LC–HRMS. The core principle of this method is the overlapping analytical scope between GC–MS and LC–HRMS (Brack et al. 2016), which allows many of the compounds identified by GC–MS to be subsequently screened for as suspects in the LC–HRMS workflow. The primary advantage of this method is that it leverages the relatively straightforward and rapid identification capabilities of GC–MS. Furthermore, because the suspect lists are derived from the same sample, this approach significantly increases the confidence of identifications in LC–HRMS analysis. An example of this strategy is the work by Su et al. (2023), who utilized a suspect list of over 200 compounds previously identified by GC–MS in the same samples to aid in the structural elucidation of features detected by LC–QTOF. This approach successfully led to the identification of several compounds, such as *N*,*N*‐dimethyldodecylamine and *N*‐phenyl‐2‐naphthylamine. The reliability of these identifications was further substantiated by the high degree of matching between the experimental mass spectra and the in silico MS/MS spectra generated by MS‐FINDER, as well as the similar distribution patterns of these compounds across samples in both the GC–MS and UPLC–QTOF–MS analysis (Figure 5).

#### 5.2.2 Multidimensional Filtering Approach in Suspect Screening

A key limitation of using large suspect lists in food packaging analysis is the high false‐positive rate when identification relies solely on exact mass and isotopic patterns. To address this, recent suspect screening workflows have been enhanced by incorporating additional filtering parameters, such as predicted RT and CCS values, and in silico fragmentation spectra (Blanco‐Zubiaguirre et al. 2020; Song, Canellas, et al. 2022b; Tisler and Christensen 2022; Tisler et al. 2024). This multidimensional approach substantially improves identification confidence by effectively reducing the number of potential candidates. For instance, Song, Canellas, et al. (2022b) identified migrants from polyamide spatula using a suspect screening approach that utilized a list of over 9000 compounds. By supplementing the primary *m/z* filter with RT and CCS data, the number of potential candidates was reduced by 75% and 29%, respectively. The combined application of these orthogonal filters ultimately eliminated approximately 83% of the candidates. This substantial reduction significantly lessens the burden of manual verification, thereby enhancing both the efficiency and confidence of compound identification.

#### 5.2.3 Inherent Limitations of Suspect Screening

While suspect screening is an efficient tool for identifying a broad range of known compounds in food packaging, it faces two inherent limitations. The first pertains to the confidence of identification. According to the confidence levels proposed by Schymanski et al. (2014), the level of identification confidence achieved by suspect screening is typically limited to Level 2 and, in some cases, may even be restricted to Level 3 due to the existence of isomers. The second limitation is the inherent inability of suspect screening to identify truly unknown compounds (i.e., “unknown‐unknowns”). Theoretically, however, this limitation can be partially overcome. Some “unknown‐unknowns,” such as the degradation products of additives, can be brought into the scope of a suspect screening workflow by augmenting the suspect list with structures predicted by transformation predictors (e.g., EnviPath, Zeneth). This predictive strategy has been successfully applied in environmental analysis to identify unknown transformation products of organic pollutants (Bijlsma et al. 2019; Rocco et al. 2022). However, to the best of our knowledge, its potential for identifying migrant transformation products in food packaging has not been fully explored.

### 5.3 NTA

NTA extends beyond the scope of targeted and suspect screening, enabling the identification of truly unknown compounds. Several applications of NTA in food packaging are presented in Table 4, which cover diverse food packaging materials and analytical platforms. Based on an in‐depth analysis of these NTA case studies and drawing upon our extensive experience in food packaging analysis, we classify NTA strategies into data‐driven and deductive reasoning approaches.

**TABLE 4:** Nontargeted analysis of migrants from food packaging.

Food packaging materials	Food simulants/extraction	Analytical techniques	Workflow	Compounds identified	Reference
100 food contact materials	Ultrasonication extraction with hexane/acetone (v/v, 1:1)	UPLC–Q‐Orbitrap	Ten characteristic fragments used for nontargeted screening of OPEs	Seven novel OPEs were identified	Wang, Xiao, et al. 2022
75 plastic food packaging materials	Ultrasonic extraction with hexane/acetone (v/v, 1:1)	UPLC–Q‐Orbitrap	Phosphite ion (PO_3_^−^, *m*/*z* 78.9591) was used as a characteristic fragment for alkyl di‐OPEs.	Eight alkyl di‐OPEs were identified	Xiao et al. 2023
Biodegradable food packaging	Tenax	UPLC–QTOF	Elemental composition calculation, ChemSpider and SciFinder searching, and MS/MS spectral interpretation	Five oligomers from adhesive, derived from the reaction between 1,4‐butanediol and adipic acid	Canellas et al. 2015
PE food packaging	10%, 50%, and 95% ethanol, 3% acetic acid, Tenax	UPLC–IMS‐QTOF	Elemental composition calculation, ChemSpider searching, and MS/MS spectral interpretation	35 compounds were identified, 17 of which were NIAS, including octylphenol ethoxylate and *N*,*N*‐bis(2‐ hydroxyethyl)alkylamines	Vera et al. 2019
Irradiated PET/PE films	95% ethanol	UPLC–QTOF	Elemental composition calculation, followed by structure analysis based on MS/MS spectra and ChemSpider searching	Radiolysis products derived from Irgafos 168 were identified	Wang et al. 2021
Plant fiber/plastic composites	Extraction with ethanol, migration (isooctane and 4% acetic acid)	UPLC–QTOF	NIST 20, home‐built MS/MS libraries. MoNA and GNPS. Manual elucidation based on exact mass, isotope ratio, characteristic fragment ions, and similarity of the spectrum	115 nonvolatile compounds were tentatively identified, including four melamine derivatives	Zhang et al. 2023
Reusable plastic sports bottles	Tap water	UPLC–QTOF	Elemental composition calculation, PubChem searching, and further verification by MS/MS spectra matching and predicted RT values	Oligomers of polycaprolactone, and a series of nonionic surfactants, PEG alkyl ethers, were identified	Tisler and Christensen 2022
Microwavable plastic food containers	Microwave and migration (10% ethanol)	UPLC–Q‐Orbitrap	Matching with mzCloud, ChemSpider, and in silico fragmentation patterns	Reaction products between photoinitiator 2‐hydroxy‐2‐methyl‐1‐phenylpropan‐1‐one and maltose	Diaz‐Galiano et al. 2023
88 food packaging materials	Ultrasonic extraction with methanol and migration test (10% and 95% ethanol)	UPLC–Q‐Orbitrap	Combined database matching, fragment library matching, and filtering based on PFAS‐specific signatures	17 PFAS were tentatively identified	Stroski et al. 2024

Abbreviations: GNPS, Global Natural Products Social Molecular Networking; MoNA, Massbank of North America; MS/MS, tandem mass spectra; NIAS, non‐intentionally added substances; NIST, National Institute of Standards and Technology; OPEs, organophosphate esters; PE, polyethylene; PEG, polyethylene glycol; PET, polyethylene terephthalate; PFAS, per‐ and polyfluoroalkyl substances; UPLC–IMS–QTOF, UPLC coupled to ion mobility spectrometry and QTOF; UPLC–Q‐Orbitrap, ultra‐performance liquid chromatography coupled to quadrupole and Orbitrap mass spectrometry; UPLC–QTOF, UPLC coupled to quadrupole time‐of‐flight mass spectrometry.

#### 5.3.1 Data‐Driven and Deductive Reasoning NTA

**FIGURE 6:**
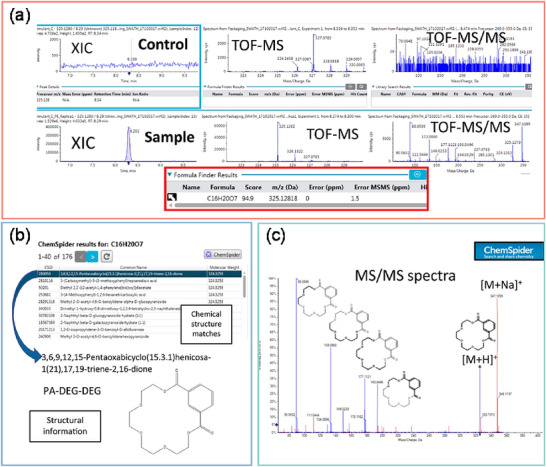
Systematic data‐driven workflow for the nontargeted identification of food packaging migrants. The figure illustrates the step‐by‐step structural elucidation of 3,6,9,12,15‐pentaoxabicyclo(15.3.1)henicosa‐1(21),17,19‐triene‐2,16‐dione using liquid chromatography coupled to quadrupole time‐of‐flight mass spectrometry (LC–QTOF). (a) Formula calculation: calculation of the potential molecular formula based on accurate *m/z* measurements and isotopic patterns. (b) Candidate retrieval: searching of the calculated formula against chemical databases (e.g., ChemSpider) to generate a list of candidate structures. (c) Structural verification: confirmation of the tentative identity via tandem mass spectra (MS/MS) spectral interpretation. Reprinted from Gomez Ramos et al. (2019) with permission from Elsevier.

The general workflow of data‐driven NTA is illustrated in Figure 6. Typically, the process begins by generating candidate elemental compositions based on monoisotopic mass and isotopic patterns. These compositions are then used to query comprehensive chemical databases (e.g., PubChem, ChemSpider) as well as specialized platforms like the NORMAN Suspect List Exchange (Mohammed Taha et al. 2022) to generate a list of candidate structures. The proposed candidate structures are subsequently evaluated by comparing the acquired MS/MS spectra of a feature against their experimental or in silico predicted fragmentation spectra (Krauss et al. 2010; Martínez‐Bueno et al. 2019). This identification process can be further enhanced by incorporating additional identification evidence, such as predicted RT and CCS values. When data‐driven approaches fail, either by yielding no candidates for an elemental composition or due to spectral mismatch of the candidates generated by database searching, a deductive reasoning NTA strategy is required. This strategy enables structural elucidation of unknowns via de novo interpretation of fragmentation patterns, which is strongly supported by a priori knowledge of the packaging material's composition, expert experience, and the integration of complementary analytical techniques (Bradley and Coulier 2007). Such processes, however, remain a formidable analytical challenge.

One of our own works integrated both data‐driven and deductive reasoning NTA to identify the chemicals in biodegradable adhesives (Canellas et al. 2015). The researchers initially employed a data‐driven approach, successfully identifying adipic acid and two biocides. However, this method failed for the four most abundant chromatographic features, as the experimental QTOF‐MS spectra showed a clear mismatch with all database‐proposed candidates. This failure prompted a pivot to a deductive reasoning strategy. Leveraging GC–MS data, the team identified two key monomers: 1,4‐butanediol and adipic acid. Based on the a priori knowledge that these monomers react to form 1,6‐dioxacyclododecane‐7,12‐dione, they hypothesized that these monomers could also form larger, structurally related lactones. This hypothesis was rigorously validated. First, the calculated monoisotopic masses of these putative neoformed compounds perfectly matched the measured *m/z* values of the unknown features. Second, the experimental fragmentation patterns were fully consistent with the proposed substructures. Although standards were unavailable, this confluence of evidence, including a plausible chemical hypothesis, identification of 1,6‐dioxacyclododecane‐7,12‐dione, 1,4‐butanediol, and adipic acid in the adhesive, exact mass confirmation, and comprehensive fragment matching, provided high confidence for the identification of these four novel neoformed compounds.

#### 5.3.2 Characteristic Fragment‐Based NTA Strategy

**FIGURE 7:**
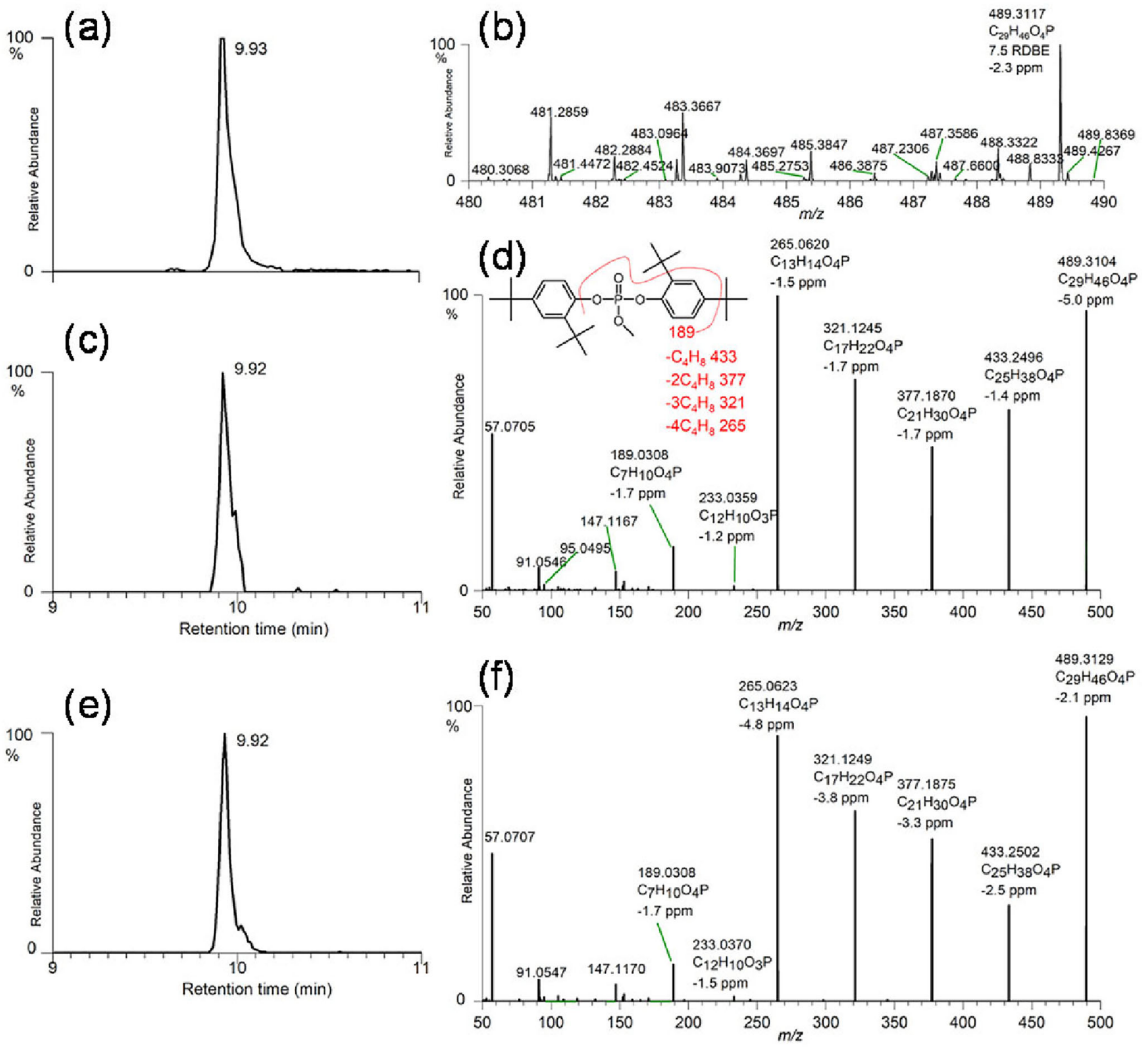
Identification of bis(2,4‐di‐tert‐butylphenyl) methyl phosphate (BDtBPMP) based on characteristic fragment‐based nontargeted analysis (NTA) strategy. (a, b) Screening phase: detection of a characteristic fragment ion (*m*/*z* 265.0624) within a specific data‐independent acquisition (DIA) isolation window (*m*/*z* 480–490), used to pinpoint the unknown precursor among multiple ions in this isolation window. (c, d) Structural elucidation: extraction of the candidate precursor (*m*/*z* 489.3127) and interpretation of its data‐dependent acquisition (DDA) tandem mass spectrum (MS/MS) to propose a tentative molecular structure. (e, f) Confirmatory phase: final verification of the identity through the precise matching of retention time (RT) and fragmentation patterns between the packaging extract and a synthesized reference standard. Reprinted from Wang, Xiao, et al. (2022) with permission from the American Chemical Society.

A characteristic fragment‐based NTA strategy represents another type of deductive reasoning approach, which has been widely used to identify novel compounds in food packaging (Bi et al. 2023; Chen et al. 2024; Wang, Xiao, et al. 2022; Xiao et al. 2023). The general workflow begins by screening MS/MS spectral data for characteristic fragments of a specific chemical class, generating multiple nontarget peaks. For each chromatographic peak, ions within the corresponding *m/z* isolation window are treated as potential precursors and undergo empirical formula prediction under predefined constraints. Ions meeting these criteria are selected as precursor ions for high‐quality MS/MS spectral acquisition. Finally, the fragmentation patterns of the obtained mass spectra are interpreted to propose structural candidates for the precursors (Wang, Xiao, et al. 2022). Figure 7 shows an example of this strategy. Wang, Xiao, et al. (2022) first screened their DIA‐MS/MS data from food packaging materials for the characteristic ion of OPEs; the search for the cresyl phenyl phosphate ion with *m*/*z* 265.0624 produced a peak in the DIA window of *m*/*z* 480−490 at 9.93 min. This precursor region initially contained 32 potential precursor ions. To filter these candidates, the researchers applied stringent constraints, including an elemental composition of C_0–100_H_0–200_O_4–20_P_1_ and a ring double bond equivalent (RDBE) value between 7.5 and 100. Only one ion, at *m/z* 489.3117 (C_29_H_46_O_4_P^+^, −2.3 ppm), satisfied these criteria. This putative precursor was then targeted for a separate DDA experiment to acquire a high‐quality MS/MS spectrum for unambiguous structural confirmation. Ultimately, interpretation of the acquired MS/MS spectrum led to the identification of a novel OPE, bis(2,4‐di‐tert‐butylphenyl) methyl phosphate (BDtBPMP). This strategy further enabled the discovery of six other novel OPEs, all of which were previously unreported in environmental or food matrices.

The efficacy of the characteristic fragment‐based NTA strategy hinges on two critical factors. First, a hybrid DIA‐DDA acquisition is recommended. The initial screening for characteristic fragments is preferably performed in DIA mode to leverage its comprehensive, high‐coverage MS/MS data, thus preventing the omission of potential fragments. Subsequently, targeted DDA is employed to acquire high‐quality, interference‐free MS/MS spectra, which are critical for unambiguous spectral interpretation and structural elucidation. Second, mobile phase optimization is essential to enhance the ionization efficiency of the target chemical class and, consequently, the abundance of their corresponding characteristic fragments. This signal enhancement can facilitate the discovery of previously obscured characteristic fragments that were originally below the detection threshold. Given that the identification of these diagnostic fragments is a prerequisite for successful characteristic fragment‐based NTA, optimizing the mobile phase has the potential to improve the discovery and structural elucidation of unknown migrants, especially for those in trace levels. As a case in point, Xiao et al. (2023) found that using an acetic acid‐based mobile phase (0.02% in both water and methanol) dramatically improved signal responses for di‐OPEs in ESI negative (ESI−) mode. Compared to a conventional 5 mM ammonium acetate buffer, this optimization yielded a 2.5‐ to 45.6‐fold increase in peak areas.

#### 5.3.3 Prioritization of Features in NTA

The most compelling advantage of NTA is its unique ability to identify novel compounds, thereby expanding the known chemical space beyond what is possible with targeted or suspect screening approaches. However, the NTA workflow is notoriously time‐ and labor‐intensive. In some instances, extensive elucidation efforts may reveal that a compound is already known in the literature or previously reported within food packaging studies. Therefore, prioritizing features of great concern that truly warrant NTA‐based structural elucidation is a critical step to efficiently allocate analytical resources. A common initial strategy is to integrate NTA with targeted and suspect screening approaches; features remaining unidentified after these initial screenings are then subjected to the more resource‐intensive NTA workflow. Subsequently, several prioritization strategies can be employed to focus on features of greatest concern.

Abundance‐based prioritization is perhaps the most common approach, as exemplified by Tisler and Christensen (2022), who selected the 50 most intense unidentified peaks for NTA‐based elucidation. However, this strategy possesses inherent shortcomings. First, signal intensity is not a surrogate for toxicity. Trace‐level components may possess higher biological activity than high‐abundance migrants. Second, as previously discussed, peak intensity is sensitive to mobile phase composition and ionization efficiency, which may introduce a bias toward compounds that are easily ionized under specific conditions. Despite these limitations, prioritizing unidentified peaks based on their intensity remains a pragmatic and widely adopted strategy, particularly when toxicological data or effect‐directed tools are unavailable.

Multivariate statistical analysis offers another approach. For instance, Canellas et al. (2022) utilized orthogonal projections to latent structures discriminant analysis (OPLS‐DA) to distinguish migrants from biodegradable teacups, focusing NTA efforts only on markers uniquely present or significantly elevated in the migration samples compared to blank tea solutions. This multivariate approach ensures that structural elucidation is restricted to specific migrants, effectively filtering out interferences from the analytical background.

Finally, effect‐directed analysis (EDA) prioritizes features based on biological activity, offering a powerful strategy to navigate the vast chemical complexity of food packaging extracts (Alvarez‐Mora et al. 2025; Bergmann et al. 2025; Sussmuth et al. 2025). The primary advantage of EDA lies in its ability to significantly streamline the NTA by focusing analytical efforts exclusively on toxicologically relevant features. For instance, Bergmann et al. (2025) recently employed a high‐performance thin‐layer chromatography (HPTLC)‐based EDA approach to screen for genotoxicants in paperboard packaging extracts. Their strategy effectively reduced the number of chemical features by at least 98% (from over 1600 to fewer than 50), allowing for the efficient identification of specific genotoxic compounds in the paperboard packaging. Similarly, Rosenmai et al. (2017) utilized EDA to pinpoint androgen receptor (AR) antagonists in sandwich wrappers. By focusing analysis solely on the bioactive fractions, they successfully identified abietic acid and dehydroabietic acid as the key contributors to the observed effects. Overall, as a prioritization strategy within the NTA, EDA ensures that the most biologically relevant features are prioritized for downstream elucidation, effectively bridging the gap between chemical analysis and biological effect.

### 5.4 Communicating Confidence in Identification

To ensure the transparency and reproducibility of identification results, it is essential to communicate the degree of confidence for each identified migrant. The most widely adopted framework is the five‐level criteria proposed by Schymanski et al. (2014), which categorizes identifications based on the cumulative weight of evidence.

**FIGURE 8:**
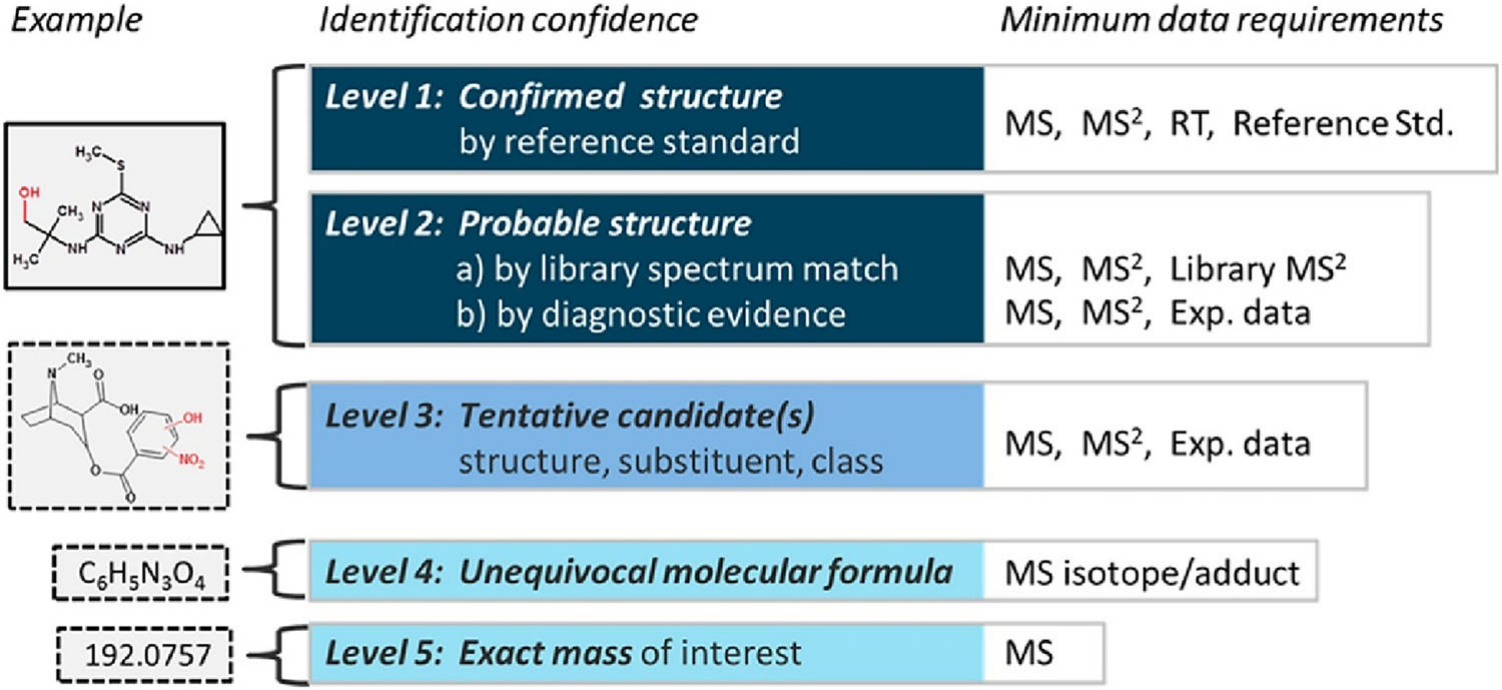
The five‐level criteria for the identification of small molecules in high‐resolution mass spectrometry analysis. MS refers to the accurate mass of the precursor ion, MS^2^ represents any form of MS fragmentation, and RT represents retention time. Reprinted from Schymanski et al. (2014) with permission from the American Chemical Society.

The classification of identification confidence and the requisite analytical data are depicted in Figure 8. In this hierarchy, Level 1 (Confirmed structure) represents the highest confidence, requiring a match of accurate mass, MS/MS spectra, and RT with an authentic reference standard. Level 2 (Probable structure) is assigned when a standard is unavailable, but a high‐confidence match is achieved via library spectra (Level 2a) or diagnostic evidence such as consistent fragmentation patterns in a homologous series (Level 2b). For many NIAS in food packaging, Level 2 often represents the highest achievable confidence due to the persistent lack of reference standards. This is exemplified by the identification of neoformed compounds in biodegradable adhesives derived from the reaction between 1,4‐butanediol and adipic acid. Although standards were unavailable, these migrants were assigned to Level 2 based on the confluence of high‐resolution exact mass, consistent fragmentation patterns, and established chemical knowledge of the polymer's monomers (Canellas et al. 2015). When MS/MS spectra match multiple candidates, a frequent challenge for isomeric migrants in food packaging materials, the identification is restricted to Level 3 (Tentative candidates), unless orthogonal data can unequivocally isolate a single structure. This is exemplified by the identification of a feature at *m/z* 413.2660 (with an RT of 8.20 min) in one of our works (Song, Canellas, et al. 2022b). The MS/MS spectra and RT for this feature are highly consistent with multiple phthalate isomers, including dioctyl phthalate, diisooctyl phthalate, and bis(2‐ethylhexyl) phthalate; an unambiguous identification remains elusive in the absence of further diagnostic evidence. Level 4 (Molecular formula) and Level 5 (Exact mass) reflect lower confidence, where only a formula or a specific mass is determined without sufficient structural evidence.

As IMS becomes increasingly prevalent in the identification of small molecules, Celma et al. (2020) introduced an enhanced five‐level confidence framework adapted specifically for IMS‐HRMS platforms. While fundamentally aligned with the five‐level criteria proposed by Schymanski et al. (2014), this framework incorporates CCS as an orthogonal parameter to complement traditional *m/z*, fragmentation, and RT data. The primary advantage of integrating IMS‐derived CCS lies in its ability to resolve isomeric species and has the potential to filter out some unlikely candidates, thereby improving the identification confidence from Level 3 to Level 2. Furthermore, Celma et al. (2020) introduced the “Level 1*” category to address instances where the RT deviates significantly due to matrix effect, yet structural certainty is maintained through the high reproducibility of CCS and MS/MS matching. This distinction is particularly advantageous for characterizing migrants in complex foodstuffs, where traditional RT‐based confirmation may lead to false negatives.

## 6 Current Bottlenecks

### 6.1 Limited Availability of Food Packaging‐Related MS/MS Libraries

Currently, only a small fraction of the features detected in food packaging analysis can typically be identified. For instance, Zimmermann et al. (2021) tentatively identified merely ∼8% of all features detected in plastic products via UPLC–QTOF. A significant limitation that hinders the high‐throughput identification of food packaging migrants via LC–HRMS is the limited availability of comprehensive mass spectral databases. While several valuable E&L‐related MS/MS libraries have been established recently, including those developed by the Nerin lab (Song, Canellas, et al. 2022a; Su et al. 2023) and Zhang et al. (2016), their scope, typically encompassing several hundred compounds, remains markedly small compared to the >10,000 chemicals potentially discovered in food packaging materials (Groh et al. 2019; Groh et al. 2021; Monclus et al. 2025). This gap persists partly due to the lack of commercially available reference standards for many FCCs, as well as the nondisclosure of some proprietary, self‐built databases. The creation of a comprehensive and high‐quality MS/MS spectral database for food packaging requires the cooperation of multiple laboratories worldwide.

### 6.2 Differences in MS/MS Spectra From Different HRMS Instruments

Even applying exactly the same experimental conditions of ionization (e.g., ESI), mobile phase, gradient, and LC column, the MS/MS spectra acquired at high collision energies can vary substantially across different commercial platforms. This variability arises because MS/MS spectra are inherently influenced by the specific geometries of the ion source and collision cell, mass analyzer configurations, and detector sensitivities (Bristow et al. 2002; Hoang et al. 2024). For instance, Hoang et al. (2024) demonstrated that relative ion intensities in high‐energy (40 eV) spectra exhibit significant variability across various HRMS platforms, thereby introducing notable spectral discrepancies and complicating cross‐platform data comparison. This fact represents an additional difficulty when comparing the fragments of a specific unknown compound obtained by other researchers.

### 6.3 Difficulty in Assignment of Ion Species

The initial step in NTA‐based identification for food packaging typically involves calculating possible molecular formulas based on exact mass measurements, isotopic patterns, and putative ion types. However, a critical bottleneck arises within this very important step: the potential misassignment of ion species, particularly the common presumption that the most intense peak in ESI positive (ESI+) mode corresponds to the protonated adduct, [M + H]^+^. As a matter of fact, the ESI process is inherently complex and frequently generates a variety of other adduct ions besides [M + H]^+^, including sodium ([M + Na]^+^), potassium ([M + K]^+^), and ammonium ([M + NH_4_]^+^) adducts (Houriet et al. 2025). Critically, when these adducts are mistakenly interpreted as [M + H]^+^ during database searches, it leads to erroneous structural annotations. A feasible method for determining ion species relies on the exact mass differences between ions observed in the MS^1^ spectrum. For example, the characteristic mass difference between the [M + H]^+^ and [M + Na]^+^ adducts is approximately 21.9819 Da. Another method for determining ion species involves analyzing analytes under both ESI+ and ESI− ionization modes. Comparing the exact *m/z* values obtained in each mode can aid in identifying the adduct type. For example, Xiao et al. (2023) employed this approach to identify a suspected aryl di‐OPE peak. They acquired MS data in both ESI+ and ESI− modes, detecting precursor ions at *m/z* 327.0776 and *m/z* 325.0640, respectively. This observation strongly indicated the formation of [M + H]^+^ and [M − H]^−^ ions for this feature. However, the applicability of both methods is constrained by specific prerequisites. The first approach is contingent upon the simultaneous formation and detection of multiple adduct ions, while the second requires that the analyte ionizes efficiently in both polarities. Neither of these prerequisites is universally met in practice.

### 6.4 Substantial Discrepancies in Identification Workflows

Currently, substantial variations characterize the identification workflows for food packaging migrants. Significant differences exist in the software (commercial vs. open‐source), data preprocessing parameters (e.g., criteria for blank subtraction, signal‐to‐noise thresholds, minimum peak intensity), and databases utilized by different research groups (Koster et al. 2025). This methodological heterogeneity often leads to considerable inconsistency in identification results across laboratories. Furthermore, the evaluation of candidate structures frequently relies heavily on expert judgment, which introduces further subjectivity and uncertainty into the identification process. Therefore, future efforts should focus on promoting the standardization and harmonization of NTA methods, particularly for NIAS.

## 7 Future Prospects

### 7.1 Collaborative Development of an Open MS/MS Library

The continued generation and public sharing of high‐quality, standard‐verified MS/MS spectra remains the cornerstone for advancing the field of food packaging analysis. This undertaking requires global cooperation among different laboratories and with manufacturers of reference standards. To facilitate this, several key elements are necessary: (1) the inclusion of spectra acquired from various instrument platforms (e.g., QTOF and Orbitrap from different vendors) to account for the inherent variability in ESI–MS/MS spectra and improve library coverage; (2) the adoption of harmonized MS/MS acquisition protocols (e.g., standardized collision energy ramps) to mitigate spectral discrepancies across different instrument platforms. Although ESI–MS/MS data inevitably vary across analytical systems, selecting appropriate collision energies can still enhance spectral consistency. For instance, Hoang et al. (2024) demonstrated that MS/MS spectra acquired with a collision energy of 20 eV across five different platforms exhibited the highest similarity, suggesting that minimizing inter‐platform MS/MS variability and facilitating more reliable data sharing are technically achievable; (3) the establishment of a centralized, open‐access repository dedicated to FCCs, supported by a unified data format that includes essential MS‐related metadata such as ionization modes, collision energies, and mobile phase systems; and (4) the strategic partnership with reference standard manufacturers to address the high cost and limited availability of pure compounds. Since spectral library construction requires only minute quantities of standards, the development of customized, multicomponent mixed standards specifically tailored for FCCs represents a cost‐effective solution. Such collaboration would alleviate the financial barriers for individual laboratories and accelerate the expansion of open‐access databases. Simultaneously, manufacturers would benefit by establishing their products as industry benchmarks and aligning their catalogs with emerging regulatory requirements.

In addition to spectra generated from standards, a complementary strategy to enrich MS/MS libraries is the disclosure of spectra for compounds identified at confidence level 2 (Probable structure). Obtaining reference standards for every potential FCCs is impractical. However, high‐confidence identifications can often be achieved even without standards by combining orthogonal evidence, such as diagnostic MS/MS fragments, RT behavior (e.g., within homologous series), and the presence of structurally related compounds. For example, Vera et al. identified a series of *N*,*N*‐bis(2‐hydroxyethyl) alkylamines in PP and polyethylene (PE) packaging (Vera et al. 2019; Vera et al. 2018). They observed a consistent mass difference of *m/z* 28.0348 (corresponding to C_2_H_4_) between consecutive homologs and a shared fragmentation pattern featuring characteristic ions (*m/z* 70.0646, 88.0768, and 102.0903). While only *N*,*N*‐bis(2‐hydroxyethyl) dodecylamine was confirmed with a standard (Level 1), the weight of evidence provided high confidence for the proposed structures of the other homologs. Similar cases involving well‐characterized homologous series are common in the literature, including the *N*,*N*‐dimethyl alkylamines reported by Su et al. (2023) and the polyethylene glycol (PEG) alkyl ether surfactants identified by Tisler and Christensen (2022). We argue that disclosing the MS/MS spectra for such high‐confidence, non‐standard‐confirmed identifications, accompanied by clarification of the confidence level, is reasonable and would significantly enrich current spectral libraries for food packaging analysis. However, this approach faces challenges, primarily the risk of propagating misidentifications and the lack of standardized curation protocols for non‐standard‐confirmed data.

To overcome these barriers, a tiered curation framework is necessary. Level 2 spectra should only be integrated into open libraries if they are accompanied by transparent identification evidence, such as homologous series or diagnostic fragments that justify the structural assignment. Furthermore, the adoption of crowdsourced curation platforms, where the community can flag or validate entries, could mitigate the risk of error propagation. By facilitating the accessibility of these high‐confidence putative spectra, the field can minimize redundant structural elucidation efforts in re‐identifying common migrants.

### 7.2 Introduction of New Techniques in Food Packaging Analysis

The adoption of novel analytical techniques is a key driver of innovation in food packaging analysis. To facilitate a comparative understanding of the evolving technical landscape, Table 5 summarizes several novel techniques, ranging from multidimensional separation to ultrafast data acquisition, highlighting their specific contributions to analytical sensitivity and identification confidence.

**TABLE 5:** Technical opportunities for improving sensitivity and identification confidence.

Opportunity area	Specific technique/strategy	Benefit to sensitivity	Benefit of identification
Advanced sampling	SPME, P&T	Higher enrichment factors compared to traditional headspace	Allow the identification of low‐trace volatiles
Multidimensional separation	GC × GC	Peak compression effect through thermal modulation leads to a dramatic increase in S/N	Structured elution patterns resolve homologous series and eliminate co‐elution interferences
Liquid phase optimization	Mobile‐phase additives (e.g., NH_4_​COOH, CH_3_​COOH)	Enhances ionization efficiency and spray stability in ESI	Promotes stable adduct formation (e.g., [M + NH4​]^+^) for unambiguous mass determination
Soft ionization	APCI	Signal concentration into molecular ions rather than fragments	Preserves molecular ion ([M]^+^ or [M + H]^+^); essential for elemental formula calculation
Gas‐phase separation	IMS	Reduction of chemical noise and matrix interferences through drift time alignment	Resolves isomers; provides CCS as a reliable identification parameter
Data acquisition	Narrow‐window DIA (nDIA)	Ultrafast scan rate (>200 Hz) allows for higher sampling density of transient trace peaks	DDA‐level spectral purity achieved via narrow isolation windows while maintaining DIA's comprehensive coverage

Abbreviations: APCI, atmospheric pressure chemical ionization; CCS, collision cross section; DDA, data‐dependent acquisition; DIA, data‐independent acquisition; ESI, electrospray ionization; GC × GC, comprehensive two‐dimensional gas chromatography; IMS, ion mobility spectrometry; nDIA, narrow‐window data independent acquisition; P&T, purge and trap; S/N, signal‐to‐noise ratio; SPME, solid‐phase microextraction.

GC × GC has emerged as a transformative tool for resolving complex mixtures that often appear as unresolved humps in conventional 1D GC, such as polymer oligomeric saturated hydrocarbons (POSH) and mineral oil saturated hydrocarbons (MOSH). By utilizing structured elution patterns, GC × GC can distinguish PE oligomers (characterized by even‐numbered *n*‐alkanes) and PP oligomers (displaying two rows of isomeric signal clusters with a distance of three carbon atoms) from mineral oil contaminants or natural waxes (Biedermann and Grob 2015, 2019). This systemic ordering facilitates accurate source attribution and the grouping of related substances for toxicological assessment.

Parallel to these chromatographic advances, as a novel separation technique, IMS is already well‐established in fields like environmental science and metabolomics. Its recognized advantages, such as the ability to distinguish isomers, generate cleaner MS/MS spectra, and reduce false positives in nontargeted studies (Asef et al. 2023; Celma et al. 2020; Foster et al. 2022; Song et al. 2023), are highly relevant to the challenges in our field. However, its application to food packaging migrants remains surprisingly limited to date (Canellas et al. 2019, 2020; Song, Canellas, et al. 2022b; Song, Dreolin, et al. 2022). Given the increasing complexity of food packaging materials, exploring the full potential of IMS to resolve key analytical challenges, such as the separation and identification of isomeric migrants, represents a critical and promising area for future research.

Complementing these separation advances, a transformative shift is occurring in data acquisition through the emergence of narrow‐window DIA (nDIA) (Kang et al. 2026). Enabled by the next‐generation Orbitrap coupled to the Asymmetric Track Lossless (Astral) platform, which integrates the Orbitrap with an Astral analyzer to achieve MS/MS scan speeds exceeding 200 Hz, nDIA represents a technical milestone in high‐throughput chemical characterization. Unlike traditional DIA, which utilizes broad isolation windows that compromise spectral purity due to chimeric interferences, nDIA employs ultra‐narrow windows (e.g., 2 *m/z*) to fragment all ions within a sample (Guzman et al. 2024). This strategy effectively converges the exhaustive coverage of DIA with the high specificity and spectral quality typically associated with DDA. By facilitating the detection of trace‐level contaminants with DDA‐level confidence, nDIA holds the potential to significantly enhance the reliability of NTA in complex food packaging matrices.

### 7.3 Combination of Multiple Techniques

Integrating multiple analytical techniques is a powerful strategy for obtaining complementary structural information on food packaging migrants. This integration can occur at various levels: at the sample pretreatment stage, by combining complementary techniques such as P&T and solvent extraction for volatile and semivolatile migrants, respectively (Vazquez Loureiro et al. 2025); at the instrumental level, by combining different platforms like GC–MS and LC–MS (Sapozhnikova 2021); at the ionization level, by using varied sources (Su et al. 2019); or at the data acquisition level, by employing different modes (Wang, Xiao, et al. 2022). Crucially, confirming an identification with an orthogonal technique that utilizes different separation and/or ionization mechanisms lends significant support to the confidence of the identification.

However, it should be noted that the practical implementation of such integrated strategies is often constrained by several factors. First, the high maintenance costs of operating multiple platforms, such as both GC–HRMS and LC–HRMS, present a significant economic barrier for many laboratories. Second, the substantial increase in analysis time and data complexity inherent in multiplatform workflows can reduce sample throughput, making them less suitable for high‐efficiency screening. Finally, the lack of standardized, automated tools for cross‐platform data fusion remains a major bottleneck, necessitating specialized expertise for the manual correlation of multiple datasets. Balancing the gain in identification confidence against these constraints remains a key challenge in the field.

### 7.4 Establishment of RI for Liquid Chromatography

The RI offers a standardized scale for chromatographic retention, overcoming the poor reproducibility of absolute RT values by normalizing analyte retention against reference standards. This conversion yields a more system‐independent value, significantly enhancing the reliability of compound identification across different experiments and laboratories. In GC–MS, RI has long served as a critical identification parameter, used alongside mass spectral data to confirm or refute putative structures (Estremera et al. 2025; Su, Vera, Salafranca, et al. 2021). However, its practical application in LC systems remains significantly constrained compared to GC. This limitation stems primarily from the lack of universal reference standards and the inherent complexity of LC systems (particularly the mobile phase), which severely compromise the transferability and intercolumn/interlaboratory reproducibility of RI values.

Despite these challenges, recent studies have begun to address this gap by developing LC–RI systems based on novel reference standards, such as *N*‐alkylpyridinium sulfonates (Stoffel et al. 2022) or cocamide diethanolamine homologous series (Aalizadeh et al. 2022). Further standardization of LC methods and the development of suitable reference standards have the potential to produce reproducible RI values. The inclusion of LC–RI matching would add additional evidence to compound identification, particularly in NTA.

### 7.5 Application of Machine Learning (ML)

ML represents a highly promising frontier for addressing persistent challenges in the identification of food packaging migrants, particularly within suspect screening and NTA workflows. Researchers are now leveraging ML to predict fundamental molecular properties that bolster identification confidence when reference standards are lacking. Recent studies have demonstrated the utility of models for predicting RT (Sapozhnikova and Nunez 2022; Song, Canellas, et al. 2022b; Taylor and Sapozhnikova 2022; Xu, Chughtai, et al. 2023), CCS (Song, Canellas, et al. 2022b; Song, Dreolin, et al. 2022), and MS/MS fragmentation spectra (Su et al. 2023). These in silico predicted values provide crucial orthogonal data, significantly assisting in candidate prioritization and the structural elucidation of novel migrants absent from existing libraries. The integration of these predictive ML tools promises to streamline NTA workflows, enhance identification accuracy, and partially circumvent the limitations imposed by the scarcity of reference standards, ultimately accelerating the chemical safety assessment of food packaging materials.

However, several barriers to the broader implementation of ML in food packaging analysis must be acknowledged. First, the predictive power of ML models is constrained by the scarcity of large and high‐quality training datasets specific to food packaging chemicals. This lack of training data often leads to limited model generalizability, particularly when encountering complex NIAS or oligomers that fall outside the chemical space of models. Second, overly stringent ML‐based filtering carries the risk of discarding true positives. If a genuine migrant's experimental properties (e.g., RT or CCS) deviate from the predicted range due to matrix effects or structural novelty, it may be erroneously filtered out, potentially leading to a false negative in suspect screening and NTA. Furthermore, implementing robust ML workflows requires high‐level cross‐disciplinary expertise in both analytical chemistry and data science, which remains a significant barrier for many laboratories. Overcoming these challenges will require the continued expansion of specialized open‐access training databases and the development of more robust, user‐friendly ML frameworks tailored for the food packaging analytical community.

### 7.6 Combination of Chemical Analysis With Bioassays

The widespread adoption of HRMS, coupled with advances in data processing software and the expansion of relevant databases, has enabled suspect screening and NTA to simultaneously identify hundreds to thousands of chemicals within complex sample matrices. This has significantly expanded our understanding of the chemical landscape in food packaging. However, this enhanced detection capability presents a new and significant challenge: how to efficiently sift through these extensive chemical lists to prioritize the truly high‐hazard compounds for subsequent risk assessment.

To address this prioritization challenge, integrating chemical analysis with toxicity testing is a powerful strategy. EDA, a well‐established approach, typically involves fractionating a sample, identifying biologically active fractions via bioassays, and then performing chemical analysis focused only on those fractions to identify the causative chemicals (Liu et al. 2024). Beyond this traditional workflow, innovative methods are emerging, such as predicting toxicological endpoints (e.g., endocrine disruption) directly from high‐resolution MS/MS data (Zhang et al. 2025). A key advantage of such predictive approaches is the potential to bypass the laborious structural elucidation of numerous compounds irrelevant to the specific toxicological target. However, the efficacy of these in silico models is inherently tied to the quality and diversity of the training data, posing a significant risk of false negatives for chemical classes that remain underrepresented in current databases (Van Bossuyt et al. 2017). Therefore, these predictive tools should be viewed as a prioritization filter within an iterative framework rather than a replacement for original experimental data. The continuous characterization of novel migrants and measurement of their toxicological data remain indispensable, as they provide the essential feedback required to refine these models and expand their predictive domain. In summary, the combination of chemical analysis and toxicity assessment, whether through established EDA or novel predictive techniques, holds great promise for the efficient prioritization and risk assessment of FCCs.

## 8 Conclusions

Despite considerable progress in analytical techniques for detecting food packaging migrants, obtaining complete and accurate chemical profiles remains a substantial challenge. Persistent bottlenecks, notably the limited availability of dedicated mass spectral databases and inconsistent identification methodologies, continue to impede both analytical throughput and data reproducibility.

Future breakthroughs will likely be driven by a synergistic approach. The enhanced integration of analytical technologies, particularly the fusion of data acquisition modes (e.g., DDA and DIA) and the incorporation of IMS with traditional LC–HRMS and GC–HRMS, will provide more robust evidence for structural elucidation. Concurrently, ML is poised to play a pivotal role in predicting molecular properties to facilitate identification when reference standards are unavailable. Progress will also depend on establishing open‐access, collaborative databases to overcome data‐sharing hurdles. Critically, a strategic shift from mere chemical identification toward hazard‐based prioritization, leveraging methods like EDA and computational toxicology, is essential to concentrate analytical efforts on the most hazardous compounds.

## Author Contributions

**Xue‐Chao Song**: conceptualization, methodology, data curation, investigation, writing – review and editing, writing – original draft, funding acquisition. **Qi‐Zhi Su**: investigation, writing – review and editing, funding acquisition. **Elena Canellas**: writing – review and editing. **Qin‐Bao Lin**: writing – review and editing, conceptualization. **Yu Zhou**: writing – review and editing. **Cristina Nerin**: conceptualization, writing – review and editing, funding acquisition.

## Conflicts of Interest

The authors declare no conflicts of interest.
